# Bionic Solar‐Powered Heavy Metal Trap for Eco‐Friendly Sludge Drying and Simultaneous Electricity Generation

**DOI:** 10.1002/advs.75516

**Published:** 2026-05-06

**Authors:** Yanlin Li, Jinglan Wang, Hailin Gu, Zhen Yu, Fawei Lin, Cunku Dong, Ningning Cao, Miao Yu

**Affiliations:** ^1^ School of Civil Engineering Tianjin Renai College Tianjin China; ^2^ School of Environmental Science and Engineering Tianjin University Tianjin China; ^3^ College of Energy Environment and Safety Engineering China Jiliang University Hangzhou China; ^4^ Department of Mechanical Engineering City University of Hong Kong Kowloon Hong Kong China; ^5^ School of Materials Science and Engineering Tianjin University Tianjin China; ^6^ College of Engineering and Applied Sciences Nanjing University Nanjing China; ^7^ School of Materials and Energy University of Electronic Science and Technology of China Chengdu China

**Keywords:** CO_2_ emission, evaporation‐induced electricity generation, heavy metal removal, interfacial solar‐driven evaporation, sludge disposal, Sustainable Development Goals

## Abstract

Sludge, a rapidly accumulating by‐product of wastewater treatment worldwide, holds great potential as an ideal coal substitute following dehydration and drying. Here, we present a solar‐powered heavy metal trap (SPHT) designed for sludge drying and concurrent electricity generation, validated using a PPy‐coated super hydrophilic wood (PPy‐H‐Wood). The SPHT in this work uniquely integrates sludge drying, heavy metal removal, and electricity generation characteristics. The indoor experiments demonstrate a rapid decrease in sludge water content from 90 % to 31 %, accompanied by an impressive 94 % reduction in free heavy metal content achieved through synergistic adsorption and evaporation. Harnessing the hydrovoltaic effect, the SPHT can generate an initial potential of 0.10 V. By monitoring this hydrovoltaic potential, the sludge drying process can be self‐detected. Furthermore, we have constructed a 20 m^2^ pilot‐scale SPHT device, achieving a sludge drying ratio of over 80 % and a heavy metal removal ratio exceeding 75 %. Life cycle assessment (LCA) indicates that replacing traditional heat drying technology with the SPHT can cut CO_2_ emissions by ∼98 %. Our work provides an eco‐friendly approach to sludge disposal and resource recovery, and may inspire more innovative solutions supportive of global Sustainable Development Goals.

## Graphical abstract

Herein, we propose a solar‐powered heavy metal trap (SPHT) for sludge disposal. The indoor experiments demonstrated that the sludge water content decreased from 90 % to 31 %, coupled with a decrease in heavy metal content to 6 % at most. Furthermore, the designed SPHT generated an initial potential of 0.10 V, and monitoring this hydrovoltaic potential enabled self‐detection of the sludge drying process.

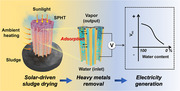

## 1 Introduction

**FIGURE 1:**
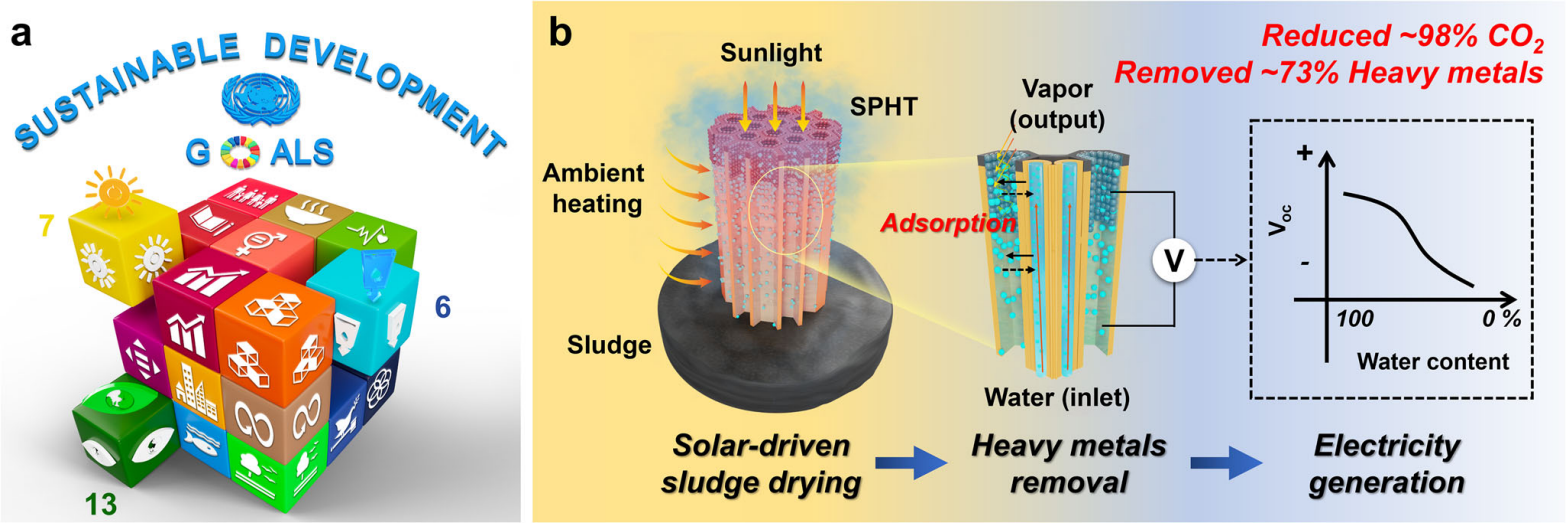
(a) Sustainable Development Goals. Specifically, 6: Clean water and sanitation, 7: Affordable and clean energy, and 13: Climate action. (b) Schematic showcasing the operating mechanism of the designed SPHT.

Water, energy, and environmental crises significantly threaten human survival, as underscored by the United Nations’ Sustainable Development Goals (i.e., the SDGs 6, 7, and 13) (Figure 1a) [1, 2]. Sludge, a by‐product of sewage treatment, contributes substantially to the above challenges, with its annual output rapidly increasing (e.g., China alone produced over 65 million tons in 2020) [3, 4, 5, 6, 7]. Indeed, sludge contains a high water content (>80 %), hazardous substances including heavy metals/pathogenic microorganisms/persistent organic pollutants, and huge calorific value after drying, highlighting its multiple characteristics of pollution, resources, as well as energy [8, 9, 10]. Eco‐friendly treatment and efficient resource utilization of sludge not only support sustainable urban water cycles but also offer potential solutions to the global environment and energy crisis [11, 12].

Sludge drying is a critical process for minimization, innocuity, and recycling, yet it presents significant energy consumption challenges along with difficulties in removing free heavy metals from sludge [13, 14]. By harnessing solar energy with minimal CO_2_ emissions, the newly emerging interfacial solar‐driven evaporation technology offers an alternative for some industrial technologies [15, 16, 17]. Recent advancements have focused on developing high‐efficiency interfacial solar‐driven evaporators for wide applications such as seawater desalination, wastewater treatment, and other energy/environment fields [18, 19, 20, 21, 22, 23]. In nature, plants extract water, nutrients, and metal ions from the soil via transpiration, a process that transports water from the soil to the air while distributing metal ions throughout the plant [24, 25, 26]. Inspired by this, specially designed evaporators have been developed to extract both water and metal ions directly from wastewater or brine, making them promising candidates for sludge drying and simultaneous heavy metal removal [27, 28, 29, 30].

Furthermore, interfacial solar‐driven evaporators are also capable of electricity generation through the hydrovoltaic effect [31, 32]. For example, a wood‐based evaporator has demonstrated an open circuit voltage (V_oc_) of 0.77 V and a short circuit current (I_sc_) of 148 µA in a 1.2 m CaCl_2_ solution [33]. Even more impressively, a living lotus has achieved a V_oc_ of 0.25 V by leveraging the transpiration process [34]. Despite these promising advances, hydrovoltaic generators are predominantly confined to liquid‐phase environments, and their applicability in solid‐liquid mixtures remains uncharted territory. Additionally, the low power output of most, if not all, of the existing hydrovoltaic systems limits their viability for decentralized energy generation or integration into large‐scale grids [35, 36]. As such, identifying suitable applications and enhancing the performance of hydrovoltaic technology are vital steps forward. The above‐mentioned challenges underscore the infancy of this field and emphasize the pressing need for intensive research and development efforts to unlock its full potential.

Herein, we propose a solar‐powered heavy metal trap (SPHT) for sludge drying and simultaneous electricity generation (Figure 1b). Our primary objective is to present the structural and functional characteristics of SPHT. We adopted a PPy‐coated super hydrophilic wood (PPy‐H‐Wood) to validate its efficacy, thanks to its exceptional anti‐pollution properties and broad‐spectrum heavy metal capture capabilities. The SPHT achieved an impressive evaporation rate of 5.13 kg m^−2^ h^−1^ (ca. 17.7 times that of sludge alone) while drying sludge with 90 wt. % water content under one sun. High‐performance indoor experiments demonstrated a rapid reduction in sludge water content from 90 % to 31 %, coupled with a significant decrease in heavy metal content to 6 %. Furthermore, the designed SPHT generated an initial potential of 0.10 V due to the hydrovoltaic effect. Most notably, monitoring this hydrovoltaic potential enabled self‐detection of the sludge drying process, which inspired a novel application for the hydrovoltaic technique. Theoretical simulations further elucidated the underlying mechanisms driving this performance. Finally, a 20 m^2^‐pilot experiment highlights the practical application potential of the SPHT. Life cycle assessment (LCA) further confirms that the SPHT is significantly more eco‐friendly than the industry benchmark, as evidenced by notably lower impact scores across all environmentally relevant metrics [24, 37].

## 2 Results and Discussion

### 2.1 Fabrication and Characterization of the designed SPHT

**FIGURE 2:**
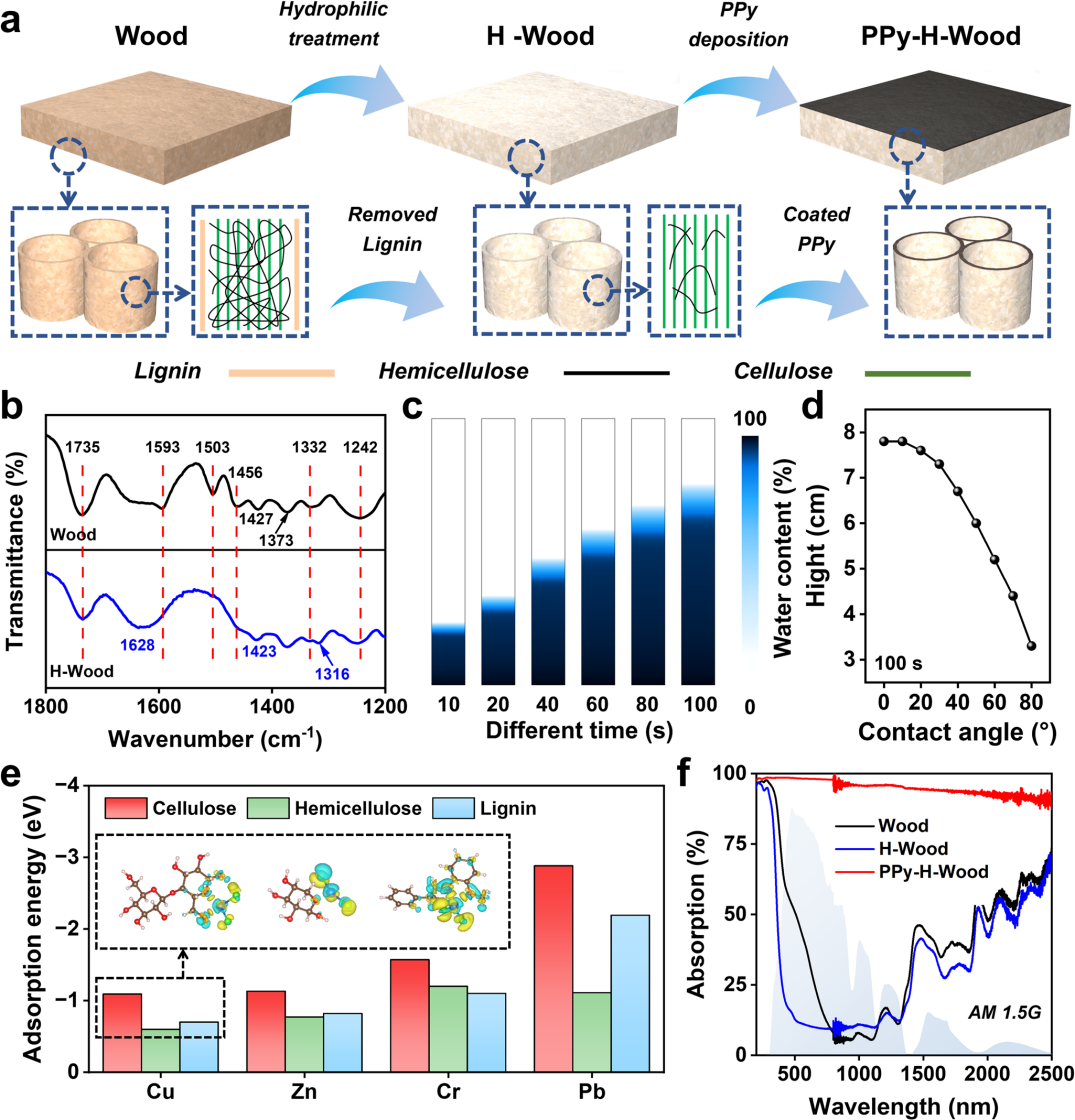
(a) Fabrication processes of PPy‐H‐Wood by delignification and PPy coating. (b) FTIR spectra of the pristine wood (Wood) and H‐Wood. (c) The water rose in H‐Wood, simulated by COMSOL software. (d) The water pumping height in wood samples with different water contact angles. (e) The adsorption energy of cellulose, hemicellulose, and lignin for different heavy metal ions. Inset: Charge density difference for cellulose, hemicellulose, and lignin. (f) UV–Vis–NIR absorption spectra of the different samples.

We selected balsawood, a widely available and cost‐effective softwood, as the substrate for fabricating the SPHT, primarily due to its abundant natural channels that facilitate the transport of water and metal ions [38, 39, 40]. To further enhance the water extraction capability from sludge, the lignin was removed from the pristine wood (Figure 2a, details in Supporting Information), thus obtaining the super hydrophilic wood (H‐Wood). This delignification process preserved the natural channels of the wood (Figure S1) along with significantly enhancing its hydrophilicity (Figure S2), reducing the water contact angle from ∼76.2° to ∼0°. Fourier‐transform infrared spectroscopy (FTIR) well confirmed the successful lignin removal, as its characteristic peaks at 1593, 1503, 1456, 1426, 1373, and 1332 cm^−1^ vanished, while hemicellulose peaks at 1735 and 1242 cm^−1^ remained (Figure 2b) [41, 42, 43, 44].

In fact, H‐Wood can offer two advantages over the pristine one: (1) Enhanced water pumping capability. COMSOL simulation was used to study this process. When the H‐Wood was placed on the sludge, the water rose ∼7.8 cm within 100 s (Figure 2c), well demonstrating its high water extraction capability; in contrast, the pristine one presented a poor water transport performance (Figure S3). These results were corroborated by experimental findings (Figure S4). Indeed, the water‐pumping ability of different wood samples is closely related to their surface water contact angle. Generally, a lower water contact angle consistently translated to improved water pumping performance (Figure 2d). (2) Superior heavy metal capture capacity. Compared to the pristine wood, the cellulose and hemicellulose were exposed on H‐Wood as demonstrated in Figure 2a. DFT calculations showed that cellulose, hemicellulose, and lignin all exhibited negative adsorption energy for different heavy metal ions, indicating the spontaneous heavy metal capture behavior (Figure 2e). Additionally, the adsorption energy of cellulose for typical heavy metals was much higher than that of lignin and hemicellulose. As such, compared with the pristine one, H‐Wood featured more heavy metal adsorption sites because a large amount of cellulose was exposed after delignification [45]. Most notably, we did not further remove the hemicellulose from H‐Wood to ensure the mechanical strength of the sample.

To enhance optical adsorption, we then deposited a PPy layer onto the H‐Wood (Figure 2a), obtaining PPy‐coated H‐Wood (PPy‐H‐Wood). The successful coating of the PPy layer was well verified by FTIR (Figure S5). Both the porous structure and super‐hydrophilicity were well maintained as compared to H‐Wood, with small PPy particles decorated on the fiber's surface (Figure S6). Later, the UV–Vis–NIR spectra (Figure 2f) indicated that PPy‐H‐Wood featured a high light absorption of 95 % across the entire solar spectrum, far superior to that of pristine wood and H‐Wood. Whereafter, we evaluated the photothermal conversion performance of PPy‐H‐Wood under one sun (Figure S7). After 1 h of irradiation, the temperature of PPy‐H‐Wood was steady at ∼51.7°C. while the temperatures of pristine wood and H‐Wood were only ∼33.8°C and ∼29.1°C, respectively. We also monitored the changes in surface temperature of dry and wet PPy‐H‐Wood under irradiation (Figure S8). Wet PPy‐H‐Wood's surface temperature change trend was similar to that of dry PPy‐H‐Wood, but with a lower temperature increasing rate and steady‐state temperature (∼34.1°C). The above results demonstrated that PPy‐H‐Wood featured competent photothermal conversion ability. The exceptional chemical stability of our designed PPy‐H‐Wood was also well evidenced. After 12 h of ongoing ultrasonic treatment, the sample barely changed even in 0.1 m HCl or 0.1 m NaOH solutions (Figure S9). In conclusion, the combination of rapid water pumping, efficient heavy metals capture, superior photothermal conversion, as well as superb chemical stability positioned the designed PPy‐H‐Wood as a desirable candidate for SPHT.

### 2.2 Indoor Solar‐Power Experiment

**FIGURE 3:**
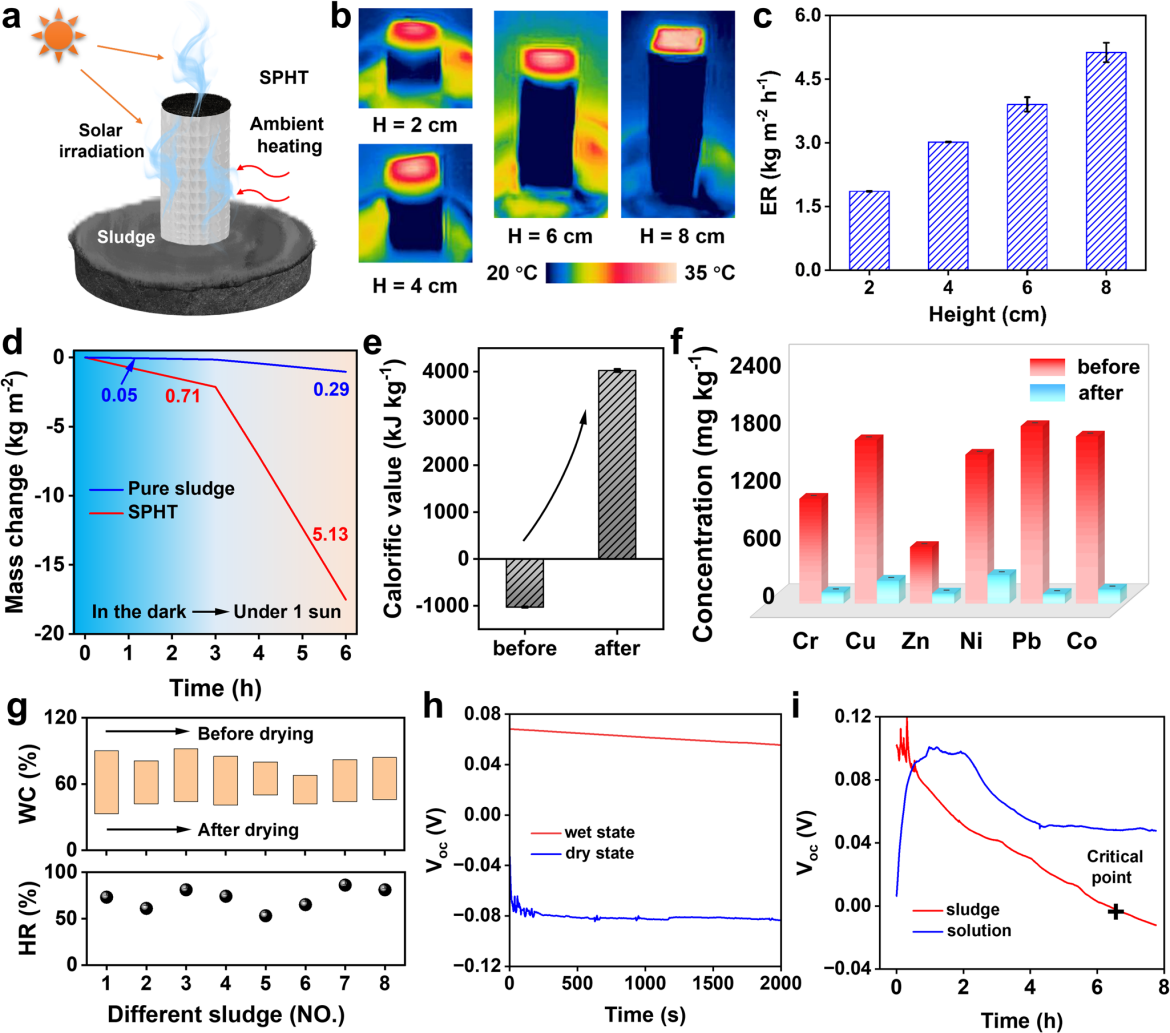
(a) Schematic of the sludge drying experiment under solar irradiation. (b) IR photos, and (c) evaporation rate (ER) of the SPHTs with different heights under one sun. (d) Mass change curves of the sludge alone and the sludge with the SPHT under one sun. (e) Calorific values, and (f) heavy metal concentrations of the actual sludge before and after drying by SPHT. (g) The water content (WC) of 8 kinds of actual industrial sludge before and after treatment by SPHT, and the corresponding heavy metal removal ratio (HR). (h) Open‐circuit potential of SPHT in the wet state and dry state, respectively. (i) SPHT surface potential in sludge and heavy metal solution (the same heavy metal concentration of solution as sludge) during the drying process.

In this part, we first compared the evaporation performance of PPy‐H‐Wood and H‐Wood in deionized water under one sun. The evaporation rate of PPy‐H‐Wood was ∼1.96 kg m^−2^ h^−1^, ca. 2.2 times that of H‐Wood (∼0.91 kg m^−2^ h^−1^) (Figure S10). The water evaporation from sludge by PPy‐H‐Wood was then investigated (Figure 3a). Within 60 min, the average temperature of the PPy‐H‐Wood top rose from 20°C to above 33°C (Figure 3b), while the PPy‐H‐Wood side walls’ temperature was lower than the ambient. Additionally, the evaporation performance was related to the sample height. As the height increased from 2 to 8 cm, the evaporation rate rose significantly from ∼1.86 to ∼5.13 kg m^−2^ h^−1^ (Figure 3c); in sharp contrast, the natural evaporation rate of sludge alone under one sun was only ∼0.29 kg m^−2^ h^−1^ (Figure 3d). However, further increasing the sample height from 8 to 16 cm resulted in a rate decline to ∼3.66 kg m^−2^ h^−1^ (Figure S11), indicating that the optimal sample height was 8 cm. This reduction in the evaporation performance for heights beyond 8 cm is likely caused by the limited water pumping capability at greater sample heights. The effect of immersion depth upon evaporation performance was further investigated (Table S1). As the immersion depth increased from 0.5 to 2 cm, the evaporation rate increased significantly, demonstrating the improved capillary‐driven water supply. However, further increasing the immersion depth to 4 cm resulted in a slight decline in evaporation rate, which can be attributed to enhanced heat loss to the bulk sludge and reduced thermal localization. Thus, unless otherwise noted, all subsequent experiments were conducted using the optimized PPy‐H‐Wood (with a sludge immersion depth of 2 cm and an evaporation height of 8 cm) as the SPHT platform.

Next, we examined the evaporation performance of this SPHT when treating sludge with varying water content. Remarkably, even when the sludge's water content decreased from ∼90 % to ∼50 %, the SPHT still maintained a decent evaporation rate of ∼4.11 kg m^−2^ h^−1^ (Figure S12). Additionally, we explored the evaporation rate under different solar fluxes. As the irradiation increased from ∼0.5 to ∼2 suns, the evaporation rate rose notably from ∼3.5 to ∼9.1 kg m^−2^ h^−1^ (Figure S13). These findings highlight that the evaporation performance can be effectively tuned by modulating the applied solar input, and the SPHT can function properly even under low‐solar‐light conditions. We subsequently conducted indoor experiments to validate the SPHT's capability for the actual sludge proposal (details in Supporting Information). After 30 h of ongoing treatment, the sludge water content reduced from ∼90 % to ∼31 %, leading to an increase in its calorific value from −1027.5 to 4023.5 KJ kg^−1^ (Figure 3e), which can be regarded as the ideal energy fuel. Notably, after the treatment mentioned above, the concentration of free heavy metals in sludge was significantly reduced to 6 %–20 % (Figure 3f), demonstrating high‐efficiency heavy metal removal along with the sludge drying. To further illustrate the universality and adaptability of our SPHT approach for sludge drying and heavy metal removal, we later tested eight different types of industrial and domestic sludge. In each instance, the SPHT performed admirably (Figure 3g). The observed variability in results across different sludge types can be attributed to factors such as viscosity, water content, and the speciation and concentration of heavy metals, each of which needs further investigation under real‐world application scenarios. Most notably, although sludges with exceptionally high heavy metal concentrations may occur in specific industrial contexts, indeed, such conditions are relatively uncommon; further evaluation under these extreme cases will thus be a focus of future studies. Additionally, for sludges with higher salinity, salt crystals may form on the SPHT surface. To mitigate potential salt fouling, a superhydrophobic surface treatment can be readily implemented [46].

We then conducted cyclic experiments for actual sludge drying over 10 cycles (28 h of evaporation per cycle, details in Supporting Information) to verify the service stability and practical application potential of the device. Throughout the 10 cycles, the average evaporation property of SPHT remained relatively stable (Figure S14a). However, the real‐time evaporation rate of SPHT gradually declined with the extended cycle time, which was primarily attributed to the sludge's progressive decrease in water content. Taking the first cycle as an example for demonstration, after ongoing running for 28 h, the evaporation rate of SPHT decreased from ∼5.3 to ∼3.9 kg m^−2^ h^−1^ (Inset in Figure S14a), while the water content of the sludge dropped to ∼41 %. However, when the used SPHT was withdrawn from the dried sludge and directly placed in the same batch of sludge with the same initial water content, the evaporation rate of SPHT can quickly return to its original value, well showcasing the designed SPHT's reusability and resilience.

SPHT's heavy metal removal performance appeared to decline since the eighth cycle, although a heavy metal removal ratio of 64 % was still achievable in the 10th cycle (Figure S14b). After 10 cycles, SPHT could be rejuvenated using an HCl solution (details in Supporting Information), thus restoring both its evaporation capability and heavy metal removal performance to their original levels (Figure S14b). Notably, the capillary effect may cause some small particles from sludge to enter the SPHT and attach to the pores at its bottom during 10‐cycle operation (Figure S15). Nevertheless, the small size of these particles did not block the macroporous structure of the SPHT or impair its water transmission and evaporation performance throughout the process. Better yet, larger particles cannot enter the SPHT because their gravity exceeds the capillary force. While it is anticipated that with further cycle operation, the bottom of the SPHT may be completely blocked by sludge particles. Fortunately, before the regeneration of SPHT, the evaporation performance did not deteriorate, suggesting that these particles did not fully clog the SPHT or obstruct its water transport performance. Beyond the regeneration, the used SPHT can also be processed into catalysts and adsorbents (Figure S16); the as‐obtained products demonstrated promising performance (Figures S17 and S18), which needs further investigation in future studies.

Since the PPy‐H‐Wood surface was rich in negative charges (Figure S19), a potential difference could be monitored when water ran through the PPy‐H‐Wood channels due to the hydrovoltaic effect [46]. This hydrovoltaic potential difference is proportional to the evaporation rate, which is determined by the sludge water content. By measuring the SPHT's hydrovoltaic potential difference, the real‐time sludge water content can be well detected (Figure S20). The dry SPHT displayed a negative surface potential, while the wet one exhibited a positive potential (Figure 3h). The average power density of wet SPHT was measured at ∼0.675 µW cm^−2^, consistent with the typical performance values early reported [47]. Because the electrical signal of a hydrovoltaic‐based electricity generator typically showed sensitivity to evaporation dynamics and environmental variations, well suggesting its potential for self‐powered sensing. Thus, we used this electrical output to detect the water content change of sludge. Throughout an 8 h evaporation process, SPHT's surface potential gradually declined from 0.10 to −0.02 V, reaching the critical point, i.e., 0 V at 6.1 h, corresponding to a water content of 32 % (Figure 3i).

We further investigated the SPHT's surface potential in a heavy metal solution under one sun. During water evaporation, it grew to 0.10 V before dropping and stabilizing to 0.05 V. The initial potential increase was likely attributed to concentration polarization at the SPHT's top. As the dispersion salinity increased from 0 % to ∼5 %, the SPHT surface potential gradually increased. However, with a further increase in the concentration at the bottom, the surface potential decreased due to the reduced Debye length (Figure S21a). Additionally, during evaporation, vapor accumulation near the top surface of the SPHT may lead to a locally elevated humidity [18, 48], which could thus contribute to the observed decrease in surface potential (Figure S21b). Due to the synergistic impact of the above two factors, the surface potential of SPHT reduced and eventually stabilized. Indeed, sludge is suitable for incineration when its water content is reduced to 40 %. Therefore, real‐time detection of sludge water content is highly necessary in terms of time and cost efficiency for practical applications, which can be achieved based on the hydrovoltaic effect, though further exploration is needed. Note that the hydrovoltaic electricity generators generally exhibited relatively low energy output due to intrinsically weak driving forces and energy dissipation associated with evaporation‐coupled processes. Therefore, rather than serving as a primary energy source, the generated electricity is better interpreted as a functional output (in this work, a self‐sensor).

### 2.3 Mechanism Exploration

**FIGURE 4:**
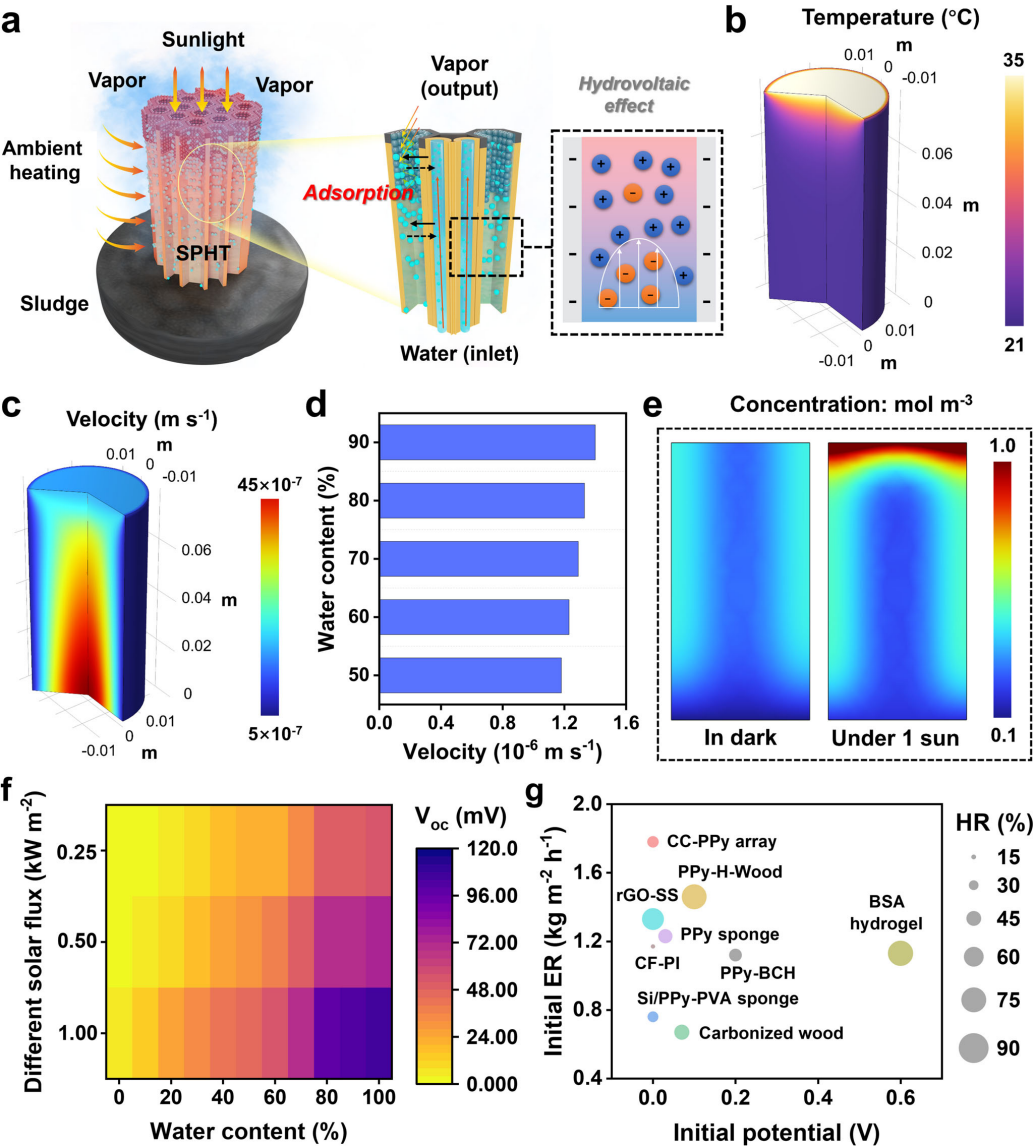
(a) The overall operation progress of SPHT. (b) Temperature distribution, and (c) velocity distribution of the SPHT simulated by COMSOL software during sludge treatment under one sun. (d) Simulated water evaporation velocity at the SPHT top when drying sludge with various water contents under one sun. (e) Simulated distribution of heavy metals in SPHT's channels for the cases under one sun and in the dark. (f) The V_oc_ of SPHT simulated by COMSOL software under different solar fluxes. (g) The performance of SPHT fabricated by different photothermal materials: rGO‐SS [52], CF‐PEI [53], BSA hydrogel [26], CC‐PPy array [54], Si/PPy‐PVA sponge [55], PPy sponge [56], Carbonized wood [57], and PPy‐BCH [58].

The operational progression of SPHT is illustrated in Figure 4a. The solution could migrate from the sludge to SPHT via capillary action in the dark, while irradiation conditions can significantly enhance solution pumping capacity by promoting surface evaporation within SPHT. As evaporation progressed, the water content within the sludge decreased. Concurrently, free heavy metal ions in the sludge were transferred to the SPHT through the combined effects of adsorption and evaporation. In addition, the negatively charged nanochannels within the SPHT device can repel hydroxide ions (OH^−^) while allowing hydrogen ions (H^+^) to pass through, which leads to the separation of positive and negative charges, thus generating a potential difference [49]. Since the amount of separated positive and negative charges is closely linked to the water transfer rate, potential changes can reflect the water content in the underlying sludge, thus, in turn, allowing for determining the endpoint of the drying process. Thus, our well‐designed SPHT effectively integrates sludge drying, heavy metal removal, and real‐time self‐detection capabilities, as elucidated below.

First, we investigated the SPHT's temperature distribution (Figure 4b) and its water transport rate (Figure 4c) under one sun using COMSOL simulations (details in Note S1). During the sludge drying process, the SPHT's top exhibited a higher temperature than its rest section; in contrast, the side walls exhibited temperatures even lower than the ambient, a finding that aligns with our experimental observations (Figure 3b). Indeed, the top surface of SPHT dominated the evaporation process due to direct solar irradiation, which provided sufficient energy input to maintain a relatively high surface temperature. In contrast, the side walls received no direct solar energy and relied mainly on limited heat conduction from the top surface. Combined with evaporative heat loss, this thus led to a net cooling effect, resulting in side‐wall temperatures lower than the ambient. In this scenario, heat localization at the SPHT's top facilitated high‐rate sludge drying; the temperature difference between the SPHT's walls and environment drove additional energy supply toward the SPHT to further promote the evaporating/drying process.

According to the simulated results (Figure 4c), the rate distribution correlated to the SPHT height, i.e., as the height increased, the water transport rate at the top gradually decreased, in good agreement with the experimental results (Figure 3c). To elucidate the mass change behavior in Figure 3d, the sludge drying process can be interpreted based on the classical evaporation theory. For sludge alone, the mass loss was slow because of the inefficient heat utilization under direct solar irradiation, where evaporation is dominated by a limited surface evaporation effect. In sharp contrast, SPHT showed a larger effective evaporation area, much more effective heat localization, and enhanced vapor pressure gradient, thus performing a higher evaporation rate. Compared with the sludge drying system alone, our SPHT also maintains a higher evaporation rate throughout the process by continuously supplying water through capillary‐driven transport and mitigating diffusion limitations. Then, we also simulated the SPHT's water transport rate when drying sludge with different water contents (Figure 4d): as the sludge's water content fell, so did the SPHT water transport rate. Overall, from an energy perspective, the absorbed solar energy within the SPHT system is more efficiently utilized for water evaporation rather than being dissipated into the surroundings, which further explains the substantial enhancement in the drying performance.

Second, we explored the SPHT's heavy metal removal mechanism. Notably, the SPHT can remove heavy metals from sludge even in the dark, albeit at a lower removal ratio than when being irradiated (Figure S22). We then applied Cu^2+^ as a typical representation of heavy metal ions to elucidate this removal process. Following the adsorption of Cu^2+^ (details in Supporting Information), the elemental mapping image exhibited a homogeneous distribution of Cu on the PPy‐H‐Wood fibers (Figure S23). X‐ray photoelectron spectroscopy (XPS) analysis provided further insights into the interaction between the SPHT and Cu^2+^ ions. Before adsorption, the C 1*s* spectra displayed major peaks at 284.2, 285.8, and 287.9 eV, corresponding to C═C/C─C, C─O, and C═O components [50], respectively (Figure S24a); while after Cu^2+^ adsorption, the peak associated with C─O vanished (Figure S24b). Similarly, in the O 1*s* spectra, three sub‐peaks were identified at 530.7, 531.6, and 532.56 eV, corresponding to C═O, O─C═O, and C─O [51], respectively (Figure S25a). Notably, the C─O peak also disappeared following Cu^2+^ adsorption (Figure S25b). Given the high affinity of heavy metal ions for phenolic and C─O groups, it is sensible that SPHT, with these functional groups, possesses inherent capabilities for heavy metal adsorption during the sludge drying process.

Accelerated water evaporation under solar irradiation could significantly enhance heavy metal removal. According to the COMSOL simulation results, the Cu^2+^ concentration in the SPHT under one sun was evidently higher than that in the dark, with concentrations peaking at the top of the SPHT compared to its rest sections (Figure 4e). This indicates that the designed SPHT is more effective at extracting Cu^2+^ from sludge when exposed to a higher solar flux (Figure S26). In addition, SPHT demonstrated robust performance in treating sludge with varying Cu^2+^ concentrations (Figure S27). Notably, the morphology of the SPHT's bottom also influenced its heavy metal removal efficiency (Figure S28). Specifically, the bionic root structure outperformed both flat and wedge‐shaped bottoms by significantly increasing the effective interface and adsorption area, thereby enhancing the metal extraction capability.

Third, COMSOL simulation was employed to study the V_oc_ of SPHT under different solar flux and water content (details were provided in Note S1). In this simulation, we assumed that the water content of the sludge remained constant throughout the whole evaporation process. Given that outdoor solar flux typically does not exceed 1 sun without a concentrator, we only calculated the V_oc_ of SPHT under irradiation conditions below this threshold. As anticipated, increasing the solar flux significantly enhanced the separation/transmission of ions, leading to a significant rise in V_oc_ (Figure 4f). Additionally, consistent with our experimental results (Figure 3i), V_oc_ gradually approached zero as the water content decreased (Figure 4f). Note that fluctuations in solar flux can also influence V_oc_, even at the same water content. Fortunately, these fluctuations do not reduce V_oc_ to zero, allowing changes in V_oc_ to still serve as an effective indicator of the sludge drying process. Nonetheless, a deeper understanding of the intrinsic relationship between outdoor weather, internal sludge factors (such as water content, heavy metal content, and charge distribution), and V_oc_ is essential. Establishing a multi‐factor database using advanced algorithms, like long‐time series analysis, is crucial in bridging the gap between scientific significance and engineering applications in the field of hydrovoltaic technology for sensing applications.

Finally, it is worth noting that the PPy‐H‐wood employed in this work serves solely as a proof of concept for fabricating the SPHT tailored for sludge disposal. Other photothermal materials with similar key attributes, like rapid water pumping, efficient heavy metal capture, superior photothermal conversion, and high chemical stability, could also be implemented for this purpose. To validate this hypothesis, we constructed nine additional SPHTs utilizing alternative photothermal materials (Figure 4g) [18, 26, 52, 53, 54, 55, 57, 58]. The evaporation, heavy metal removal, and power generation processes involved coupled heat and mass transfer, as well as electrochemical effects, making a quantitative unified evaluation highly complex. Therefore, evaporation rate (kg m^−2^ h^−1^), heavy metal removal efficiency (%), and output power density (µW cm^−2^) were used as key indicators to qualitatively assess the corresponding system performance. Certain materials, such as rGO‐SS [52] and CF‐PEI [53], lacked the above properties, resulting in relatively poor performance. In contrast, others, like BSA hydrogel [58] and CC‐PPy array [54], exhibited superior efficiency, even surpassing that of our PPy‐H‐Wood. The performance difference needs further investigation in the future. Indeed, besides the energy consumption and performance trade‐offs, a comprehensive multi‐objective optimization of the performance parameters will be the focus of future studies, too. It should be noted that the primary purpose of including these materials was not to provide a rigorous comparative evaluation, but rather to showcase the general applicability and adaptability of the proposed SPHT strategy across different materials.

### 2.4 Pilot‐Scale Real‐World Applications Evaluation

**FIGURE 5:**
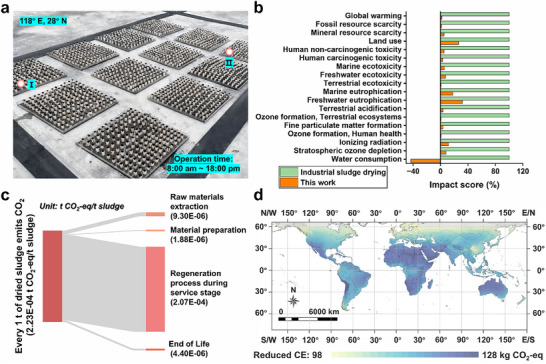
(a) Digital photo for pilot‐scale sludge drying device based on our proposed SPHT strategy. The details of locations (I) and (II) are shown in Figure S29. (b) Impact scores of all environmentally relevant descriptors for the traditional industrial sludge drying (HD) method and this newly designed green sludge drying technique based on SPHT across the life cycle, based on a functional unit of drying 1 t of sludge per day. The environmental impact in each category is normalized. (c) Carbon flow diagram of drying every 1 t of sludge by using the SPHT device throughout the life cycle. (d) Global CO_2_ emissions reduction by replacing HD with SPHT for sludge drying.

To further evaluate the real‐world applicability of SPHT, we constructed a ∼20 m^2^ pilot‐scale sludge drying device based on SPHT (Figure 5a; Figure S29) and conducted 40‐day outdoor experiments (details in Supporting Information). During the 40‐day experiments, we processed ∼15.6 tons of sludge in total (details in Supporting Information). Weather conditions during the above experiment were documented in Figure S30. The sludge drying ratio of this device exceeded 80 %, with a heavy metal removal ratio surpassing 75 % (Figure S31). Notably, while indoor trials under one sun required 28–30 h to treat the same sludge, outdoor trials (∼0.6 sun) took 30–40 h, suggesting the enhanced performance of the pilot‐scale system under outdoor conditions. The promoted outdoor performance was very likely driven by the wind‐induced convection. These results underscore the great potential of our SPHT for large‐scale, real‐life applications.

The relative environmental impacts are also a crucial factor in assessing the practical feasibility of SPHT. Here, the “cradle‐to‐grave” life cycle assessment (LCA) metric was employed to quantitatively compare the environmental performance of our rationally designed SPHT with the benchmark (i.e., the traditional industrial sludge drying method). The functional unit for this comparison was drying 1 t of sludge per day. Details of the calculation, which utilized the Simapro and Ecoinvent databases, are provided in Note S2. From Figure 5b and Note S2, one can clearly see that our SPHT strategy is far more eco‐friendly than the benchmark, as reflected in the substantially lower impact scores of all environmentally relevant descriptors. Moreover, a carbon flow diagram of drying every 1 t of sludge by using the SPHT device across the life cycle was constructed (Figure 5c), and the main influencing factors were also analyzed. The analysis reveals that SPHT only emitted ∼2.23 × 10^−4^ t CO_2_‐eq when drying 1 t of sludge per day, and optimizing its regeneration process in the service stage can further notably reduce this value. To more comprehensively and systematically show the CO_2_ reduction potential of SPHT compared to the benchmark in large‐scale global deployment, we then created a CO_2_ emission reduction map (Figure 5d). 98.2–128.4 kg of CO_2_ emissions would be avoided when drying 1 t of sludge daily, which can thus benefit the global carbon neutrality process.

Except for LCA, a detailed economic analysis is necessary to further strengthen the practical relevance of our proposed SPHT strategy. Before the formal accounting, it is worth noting that a comprehensive cost assessment typically depends on multiple factors, such as system scale, material cost, and local operational conditions, which are beyond the scope of the present work. Nevertheless, the SPHT is expected to demonstrate economic advantages due to its solar‐driven operation (i.e., minimal external energy input) and simple structure. And the future work can conduct more techno‐economic analysis. Noted that the residual ion concentration of sludge after treatment may not fully meet the standards required for direct environmental discharge. SPHT was not designed as a standalone technology for complete sludge disposal. Instead, it aimed to achieve efficient sludge drying accompanied by heavy metal reduction, thereby lowering environmental risks. The final product is dried sludge, which is more suitable for subsequent handling and disposal. More importantly, LCA results demonstrated that SPHT notably reduced both energy consumption and environmental burden compared to the conventional one. Thus, the main contribution of this work lies in providing a sustainable, eco‐friendly, and low‐energy pathway for sludge management.

## 3 Conclusions

We proposed an eco‐friendly solution for sludge disposal using a solar‐powered heavy metal trap (SPHT) strategy for the first time. The designed SPHT integrates sludge drying, heavy metal removal, and drying process self‐detection functions. Indoor experiments showed that SPHT effectively reduced the water content of sludge from ∼90 % to ∼31 %, while increasing its calorific value from −1027.5 to 4023.5 KJ kg^−1^, which can be regarded as the ideal energy fuel. Better yet, by synergizing high‐efficiency water evaporation with metal ion adsorption, this system achieved a heavy metal reduction of up to ∼94 %. Besides, by harnessing the hydrovoltaic effect, the SPHT generated an initial potential of 0.10 V. Monitoring this hydrovoltaic potential enables self‐detection of the sludge drying process. A 20 m^2^ pilot‐scale sludge drying device based on SPHT, installed outdoors, demonstrated an over 80 % sludge drying ratio and a heavy metal removal efficiency exceeding 75 %. Notably, the CO_2_ emissions of this green sludge disposal technique were notably decreased (by up to 98 %) compared to developed technologies. Altogether, our well‐designed SPHT can effectively address the major issues associated with sludge disposal, thus contributing to the grand challenges of water scarcity, energy shortages, as well as climate change.

## Author Contributions

Zhen Yu, Ningning Cao, and Miao Yu conceived the initial idea. Yanlin Li and Zhen Yu conducted the experiments. Jinlan Wang and Hailin Gu helped in formal analysis and simulation calculations. Fawei Lin and Cunku Dong contributed to the formal analysis. Zhen Yu, Ningning Cao, and Miao Yu supervised this research.

## Conflicts of Interest

The authors declare that they have no competing interests.

## Supporting information




**Supporting File**: advs75516‐sup‐0001‐SuppMat.docx.

## Data Availability

The data that support the findings of this study are available from the corresponding authors upon request.
