# Supplementary material for: Bionic Solar‐Powered Heavy Metal Trap for Eco‐Friendly Sludge Drying and Simultaneous Electricity Generation
Source: Adv Sci (Weinh). 2026 May 6;13(41):e75516. doi: 10.1002/advs.75516 (PMC13335737; doi:10.1002/advs.75516)
Supplement: Supplementary file 1 — Supporting File: advs75516‐sup‐0001‐SuppMat.docx. [file ADVS-13-e75516-s001.docx]

*Supporting Information for*

**Bionic Solar-Powered Heavy Metal Trap for Eco-Friendly Sludge Drying and Simultaneous Electricity Generation**

Yanlin Li^1, 2^, Jinglan Wang^2,^ *, Hailin Gu^3^, Zhen Yu^4,^ *, Fawei Lin^2^, Cunku Dong^5^, Ningning Cao^6,^ *, Miao Yu^7,^ *

1. School of Civil Engineering, Tianjin Renai College, Bo Xue Yuan, Tuanbo New City, Tianjin 301636, China.

2. School of Environmental Science and Engineering, Tianjin University, Tianjin 300354, China.

3. College of Energy Environment and Safety Engineering, China Jiliang University, Hangzhou 310018, China.

4. Department of Mechanical Engineering, City University of Hong Kong, 83 Tat Chee Avenue, Kowloon, Hong Kong, China.

5. School of Materials Science and Engineering, Tianjin University, Tianjin 300354, China.

6. College of Engineering and Applied Sciences, Nanjing University, Nanjing 210093, China.

7. School of Materials and Energy, University of Electronic Science and Technology of China, Chengdu 611731, China.

* Corresponding Authors

E-mail: wangjinglan@tju.edu.cn; zhenyu@cityu.edu.hk; [caonn1991@nju.edu.cn](mailto:caonn1991@nju.edu.cn); [miaoyu_che@hit.edu.cn](mailto:miaoyu_che@hit.edu.cn)

**1. Supplementary Methods**

**1.1 Fabrication of H-Wood**

H-Wood was prepared through an acid treatment method.^[1]^ Balsawood was bought from the Alibaba Platform (China). Typically, sodium chlorite (14.85 g) and glacial acetic acid (3 mL) were added into deionized water (300 mL) successively to prepare Solution A for delignification of balsawood. Note that the pH of Solution A was between 4.5 and 4.8. Balsawood with different heights was immersed into the as-prepared Solution A, and then treated at 100 °C for 12 h. H-Wood was obtained after being washed with deionized water until neutral. All reagents were used as received without further purification.

**1.2 Fabrication of PPy-H-Wood and SPHT**

PPy-H-Wood was prepared *via* a simple coating method.^[2]^ Typically, ammonium persulfate (1.14 g) and pyrrole (0.35 mL) were dispersed into deionized water (50 mL) under ultrasonic, respectively, to prepare the Solutions B and C. H-Wood (obtained from the step 1.1) was transferred to the culture dish. Solution B (10 mL) was dropwise added onto the top surface of H-Wood. Later, solution C (5 mL) was added to the top surface of H-Wood, drop by drop. After reacting for 10 min, PPy-H-Wood was obtained after being washed with deionized water. All reagents were used as received without further purification. Most notably, PPy-H wood with an evaporation height of 8 cm (total height of 10 cm) was used for the proof-of-concept of SPHT.

**1.3 Material characterizations**

H-Wood and PPy-H-wood both need freeze-drying before characterizations. The morphology was characterized by a scanning electron microscope (SEM, ZEISS Gemini SEM 300, Germany). Fourier-transform infrared (FTIR, Frontier Optica PerkinElmer, United States) spectra were obtained at room temperature in the range of 4000 to 500 cm^−1^. The contact angles were measured through a dynamic contact angle tester (OCA20, Germany). Solar absorption performance was analyzed *via* a UV-Vis-NIR spectrophotometer (UV-3101, Japan) equipped with an integrating sphere. Element distribution was tested by an X-ray photoelectron spectrometer (XPS, PHI Quantera SXM spectrometer, Germany) with a monochromatic Al Kα X-ray source.

**1.4 DFT calculation**

Density functional theory (DFT) calculations were carried out to study adsorption performance.^[3]^ The projected augmented wave (PAW) potential was employed to describe the core electrons. Most notably, the calculations were performed within the framework of the generalized gradient approximation (GGA), specifically using the Perdew-Burke-Ernzerh (PBE) functional to calculate the exchange-correlation energy.^[4]^ The plane wave basis set was extended with a cut-off energy of 500 eV. A 7 × 7 × 7 Monkhorst Pack k-point grid was utilized for structure optimizations and electronic structure calculations. The conjugate gradient method was employed to fully optimize the positions of the system until all forces on each atom were reduced to less than 0.02 eV/Å. The energy convergence of the whole self-consistent process was determined based on 10^–5^ eV. The Gaussian smearing broadening was set to 0.05 eV.^[5]^

**1.5 Solar-powered sludge drying experiment**

The indoor sludge drying experiments were conducted using a homemade optical system, and the evaporation performance of SPHT based on PPy-H-Wood was measured according to the previously reported work,^[2, 6]^ where the SPHT was put on the sludge instead of water in this case. During the measurement, a single PPy-H-Wood was placed in 60 g of sludge, in which the height of the PPy-H-Wood inside the sludge was 2 cm, and the height above the sludge varied from 2 to 16 cm. PPy-H-Wood with an evaporation height of 8 cm (total height of 10 cm) was used as the SPHT. The container’s non-PPy-H-Wood portion was wrapped to lessen the impact of the residual sludge evaporation. The evaporation rate of SPHT was calculated by the mass loss of the container before and after irradiation, similar to that of a single water system.^[7]^ Note that the determination of a local evaporation rate at the top/middle/bottom surface was technically challenging and may not be sufficiently reliable. This was because the evaporation process is inherently coupled with complex heat and mass transfer, making it difficult to accurately isolate and quantify a purely local evaporation rate. Therefore, the evaporation performance was evaluated based on the overall mass loss, consistent with the calculation method for a single water system.^[2, 6, 7]^

The sludge was ordered from the Jinnan Sewage Treatment Plant (Tianjin, China). The water content of the sludge was regulated by changing the quality of the dry sludge and water. The calorific value of sludge was determined by an oxygen bomb calorimeter (Kaiyuan Instrument 5E-AC8018, China). The heavy metal concentrations in the sludge before and after drying were measured after treatment *via* acid digestion using an inductively coupled plasma mass spectrometer (ICP-MS). Most notably, the universality evaluation of our SPHT was conducted in eight different types of industrial and domestic sludge. The various types of sludge were ordered from Jinnan Sewage Treatment Plant (Tianjin, China), Shanghai Zhuyuan Sludge Treatment Plant (Shanghai, China), and Zhejiang Sanlian Sludge Treatment Company (Hangzhou, China), respectively. The total content of free heavy metals of these sludges was shown below, which were within the typical ranges reported for both industrial and domestic sludge. Thus, the selected sludges were representative of practical conditions.

| **Number** | **Total content (mg kg^–1^ wet sludge)** | **Number** | **Total content (mg kg^–1^ wet sludge)** |
| --- | --- | --- | --- |
| No. 1 | 630.2 ± 24.1 | No. 5 | 1928.9 ± 15.8 |
| No. 2 | 547.2 ± 17.2 | No. 6 | 733.2 ± 2.2 |
| No. 3 | 195.2 ± 19.5 | No. 7 | 109.6 ± 8.3 |
| No. 4 | 324.6 ± 14.7 | No. 8 | 120.2 ± 7.7 |

The heavy metal removal ratio was calculated below:

| $\text{Heavy metal removal ratio =}\text{1}\text{–}\frac{\text{C}_{\text{t}}}{\text{C}_{\text{0}}}$ | (S1) |
| --- | --- |

where $\text{C}_{\text{0}}$ and $\text{C}_{\text{t}}$ were the heavy metal content of the sludge before and after the drying experiment (mg kg^–1^).

**1.6 Cycle experiments for sludge drying and the universality** **evaluation**

Cycle experiments for sludge drying were carried out indoors. The initial water content of the sludge was about 90%. The sludge was collected from the Jinnan Sewage Treatment Plant (Tianjin, China). Before the simulated sunlight was turned on, SPHT was placed in the sludge for 12 h to achieve adsorption equilibrium. After that, the sludge drying experiment was conducted under one sun. After 28 h of evaporation, SPHT was removed from the dried sludge and directly transferred to the same batch of sludge that was used in the first cycle with the same initial water content. The following cycle started after 12 h of standing. The method for measuring evaporation performance was similar to that described in **Section 1.5**. The evaporation performance of SPHT in every cycle was recorded. The sludge before and after each cycle was stored to test the heavy metal content. After 10 cycles, SPHT was refreshed with an HCl solution.^[8]^ Specifically, SPHT was soaked in HCl solution (10 mmol  L^−1^, 40 mL) for 12 h, and then transferred to deionized water (50 mL) for 24 h. The regenerated SPHT was then obtained for the next usage.

**1.7 Heavy metal adsorption experiments**

Heavy metal adsorption experiments involved two parts. The first part served as the control group for the indoor and outdoor experiment: SPHT was inserted into the sludge (the same setting used in the above-described indoor experiment). After indoor and outdoor experiments, the SPHT was taken out from the sludge. The heavy metal concentration in the sludge before and after adsorption was measured after acid digestion using ICP-MS. The second part was adopted to analyze the heavy metal removal mechanism from the sludge: clean sludge was blended with a 0.1 wt% CuCl_2_ solution to prepare sludge with a water content of 90%. SPHT was inserted into the sludge, in which the height of PPy-H-Wood inside the sludge was 2 cm and the height above the sludge was 8 cm. Thirty hours later, the SPHT was collected, cleaned, and dried. The distribution of heavy metal ions in SPHT was studied using elemental mapping and XPS. The sludge was always kept in the dark avoiding light in these experiments.

**1.8 Pilot-scale sludge drying experiments**

Pilot-scale sludge drying experiments were conducted in Quzhou (Zhejiang Province). Specifically, such test was conducted for 93 days, of which 40 days were selected for analysis. The daily data was collected by the meteorological station. Two main issues need to be emphasized:

1. The water content and heavy metal content of the sludge were tested every 4 days, and SPHT was used as it was over the 93 days without being refreshed.
2. When the sludge’s water content was less than 40%, the drying process was regarded as over. At this time, we used the pristine sludge to replace the dried one to complete the whole pilot experiment.

The drying ratio was calculated below.

| $\text{Drying ratio =}\frac{\text{S}_{\text{t}}-\text{S}_{\text{s}}}{\text{S}_{\text{0}}-\text{S}_{\text{s}}}$ | (S2) |
| --- | --- |

where $\text{S}_{\text{0}}$ and $\text{S}_{\text{t}}$ were the water content of the sludge before and after the drying experiment (%); $\text{S}_{\text{s}}$ was the set final water content of the sludge (40%).

The pilot experiments were conducted in 12 reactors, with a total area of about 1.3 m × 1.3 m; the sludge height in each device was about 6 cm. Given that the density of the sludge used was about 1.3 kg m^–3^, it can be deduced that the single-cycle sludge processing capacity was approximately 1.56 tons. Over a period of 40 days, we processed 10 batches of sludge, amounting to ~15.6 tons in total during the pilot experiments.

**1.9 Real-time detection of the sludge water content**

The clean sludge was blended with a 0.1 wt% CuCl_2_ solution to prepare sludge with a water content of 90%. SPHT was inserted into the sludge. During the measurement, a single PPy-H-Wood was placed in 60 g of sludge, in which the height of the PPy-H-Wood inside the sludge was 2 cm and the height above the sludge was 8 cm. The electrodes were localized at the SPHT top and the bottom near the sludge, respectively. The open-circuit voltage (V_oc_) was measured through a CHI1400 electrochemical workstation (CHI, China). Most notably, all above experiments were carried out using the gold-plated copper electrodes to avoid the effect of the corrosion potential. The potential of SPHT was measured in real time. The initial ambient humidity was approximately 33%, and the ambient humidity changed naturally. The potential changes of the SPHT in a 0.1 wt% CuCl_2_ solution were recorded as a control. The test method was the same as that for sludge alone.

**1.10 The universality evaluation of SPHT based on different materials**

First, we fabricated the nine different SPHTs based on the previous work, which are all 2D evaporation structures.^[2, 6b, 7, 9]^ The sludge disposal and electricity generation of the nine as-prepared SPHTs were measured under one sun. The sludge was collected from the Jinnan Sewage Treatment Plant (Tianjin, China). Note that, the measurement procedure was the same as described above.

**2. Supplementary Note 1:** **COMSOL Simulations**

**2.1 Water pumping process within the SPHT**

Initially, a simulated 2D model featuring identical size parameters to pristine wood and H-Wood was constructed. The phase transfer within the pristine wood or H-Wood can be described below:

| $\frac{\text{∂}}{\text{∂t}}\text{(}\text{ε}_{\text{p}}\text{ρ}_{\text{i}}\text{s}_{\text{i}}\text{)}\text{+}\text{∇}\text{·}\text{(}\text{–}\text{ρ}_{\text{i}}\text{κ}\frac{\text{κ}_{\text{ri}}}{\text{μ}_{\text{i}}}\text{(}\text{∇}\text{p}_{\text{i}}\text{–}\text{ρ}_{\text{i}}\text{g}\text{))}\text{=}\text{Q}_{\text{i}}$ | (S3) |
| --- | --- |

where $\text{ε}_{\text{p}}$was the porosity of the pristine wood and H-Wood; $\text{κ}$ and $\text{κ}_{\text{ri}}$were the permeability and relative permeability, respectively; $\text{ρ}_{\text{i}}$ and $\text{s}_{\text{i}}$ were the density and volume fraction of phase *i*; $\text{μ}_{\text{i}}$ and $\text{p}_{\text{i}}$ were the dynamic viscosity and the pressure.

Since the sum of the volume fractions of the two phases is 1, the remaining volume fraction can be calculated as follows:

| $\text{s}_{\text{1}}\text{=}\text{1}\text{–}\text{s}_{\text{2}}$ | (S4) |
| --- | --- |

The calculated capillary pressure (*P_c_*) is a function of the wet phase saturation$\text{s}_{\text{w}}$ (*i.e*., *s*_2_ in this model) and the inlet capillary pressure (*P_ec_*). Based on the Brooks-Corey model, *P_c_* is calculated as follows:

| $\text{p}_{\text{c}}\text{=}\text{p}_{\text{ec}}\frac{\text{1}}{{\text{(}\bar{\text{s}_{\text{w}}}\text{)}}^{\text{1}\text{/}\text{λ}_{\text{p}}}}$ | (S5) |
| --- | --- |

where $\text{λ}_{\text{p}}$was the porosity distribution index.

Combining Darcy’s law with the continuity equation to describe the water pumping process of SPHT:

| $\frac{\text{∂}}{\text{∂t}}\text{(}\text{ρ}\text{ε}_{\text{p}}\text{)}\text{+}\text{∇}\text{·ρ}\text{(}\text{–}\frac{\text{κ}}{\text{μ}}\text{(}\text{∇}\text{p}\text{–}\text{ρ}_{\text{i}}\text{g}\text{))}\text{=}\text{0}$ | (S6) |
| --- | --- |

where $\text{ρ}$and $\text{μ}$ were the average value of the density and the dynamic viscosity, respectively.

**2.2 Temperature distribution and water transport of SPHT**

Initially, a simulated 3D model with identical size parameters to PPy-H-Wood was constructed. Note that the evaporation driving force at the side surfaces is significantly weaker than that at the top, and thus their contribution to the overall evaporation process is secondary and can be reasonably neglected in the simulation. Given the water confinement in the capillary channels, the heat transfer in the SPHT can be described below:

| $\text{Q}\text{+}\text{∇}\text{·}\text{(}\text{k}_{\text{eff}}\text{·}\text{∇}\text{T}\text{)}\text{=}{\text{(}\text{ρ}\text{C}_{\text{p}}\text{)}}_{\text{eff}}\frac{\text{∂T}\text{(}\text{x, t}\text{)}}{\text{∂t}}\text{+}\text{ρ}\text{C}_{\text{p}}\text{v·}\text{∇}\text{T}\text{(}\text{x, t}\text{)}$ | (S7) |
| --- | --- |
| ${\text{(}\text{ρ}\text{C}_{\text{p}}\text{)}}_{\text{eff}}\text{=}\text{θ}_{\text{p}}\text{ρ}_{\text{p}}\text{C}_{\text{p, P}}\text{+}\text{(1}\text{–}\text{θ}_{\text{p}}\text{)}\text{ρ}\text{C}_{\text{p}}$ | (S8) |
| $\text{k}_{\text{eff}}\text{=}\text{θ}_{\text{p}}\text{k}_{\text{p}}\text{+}\text{(1}\text{–}\text{θ}_{\text{p}}\text{)}\text{k}$ | (S9) |

where *Q* was the solar energy absorbed by the SPHT; *T*(*x*, *t*) was the local temperature, in which *x* and *t* are the space vector and time, respectively; $\text{θ}_{\text{p}}$, $\text{ρ}_{\text{p}}$, and $\text{C}_{\text{p, P}}$ were the volume fraction, density, and the thermal capacity of the dry PPy-H-Wood, respectively; $\text{v}$, $\text{ρ}$, and $\text{C}_{\text{p}}$ were the flow speed, density, and the thermal capacity of the water, respectively; $\text{k}_{\text{eff}}$ was the effective thermal conductivity of the wet PPy-H-Wood; $\text{k}_{\text{p}}$ and *k* were the thermal conductivity of the PPy-H-Wood and water, respectively.

A steady model conducted by COMSOL Multiphysics was used to study the temperature distribution of SPHT. A constant heat flux of 1 kW m^−2^ (one sun) was applied on top, corresponding to the solar energy input on the surface of the SPHT. To carry out a qualitative analysis, the temperatures of the environment and water were set to 25 °C; the balanced heat flux was one sun.

To explore water flow within the microstructure scope, a simulated 3D model featuring identical size parameters to PPy-H-Wood was constructed. Given the water confinement of the capillary channels, the water flow within the SPHT can be described by the following equation:

| $\text{ρ}\text{(}\text{v·}\text{∇}\text{)}\text{v=}\text{∇}\text{·}\text{(}\text{–}\text{pI+K}\text{)}\text{+F}$ | (S10) |
| --- | --- |
| $\text{ρ}\text{∇}\text{·}\text{(}\text{v}\text{)}\text{=}\text{0}$ | (S11) |
| $\text{K=μ}\text{(}\text{∇}\text{v}\text{+}{\text{(}\text{∇}\text{v}\text{)}}^{\text{T}}\text{)}$ | (S12) |

where *I*, *K*, and *F* were the second-order unit tensor, viscous stress tensor, as well as gravity of water, respectively. A steady model conducted by COMSOL Multiphysics was employed to study the water transport of SPHT.

**2.3 Adsorption process**

Two models were established to study the adsorption process by using the COMSOL Multiphysics software.^[10]^

*(A) A transient model was employed to study the adsorption process of the PPy-H-Wood under one sun and in the dark.*

Before the simulations, we made the following assumptions: 1) One of the channels in PPy-H-Wood was selected to study the adsorption process; 2) The classical heat and mass transfer schemes, such as the continuity equation, were valid at this scale. A 2D geometric model was established as follows: Part A was the fiber of the PPy-H-Wood, and Part B was the channels for the water transfer and vapor escape. The details can be found in our early reported work.^[7]^


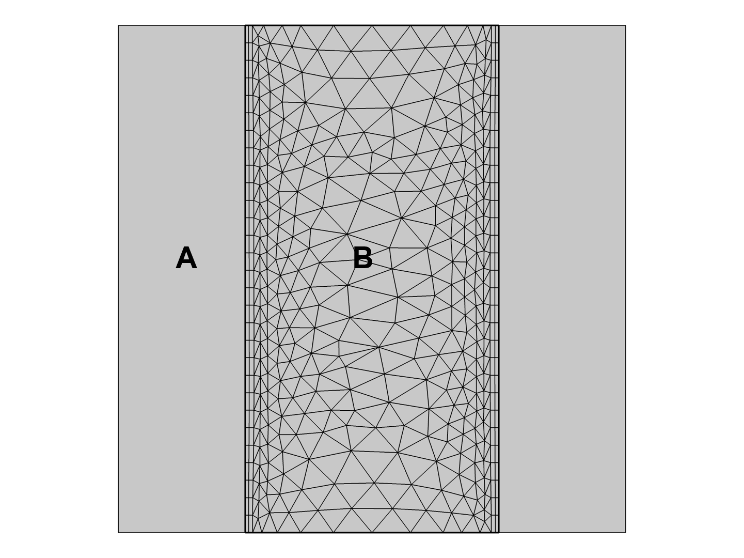


*(B) A transient model was employed to study the effective remediated area of the PPy-H-Wood with different shapes*.

Three 2D geometric models were established as follows:

*
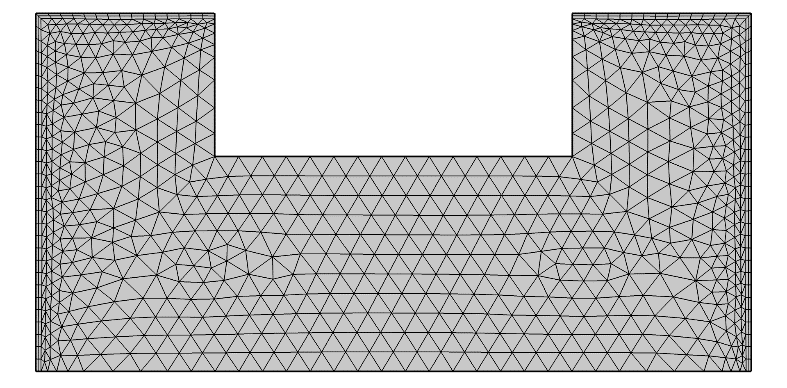
*
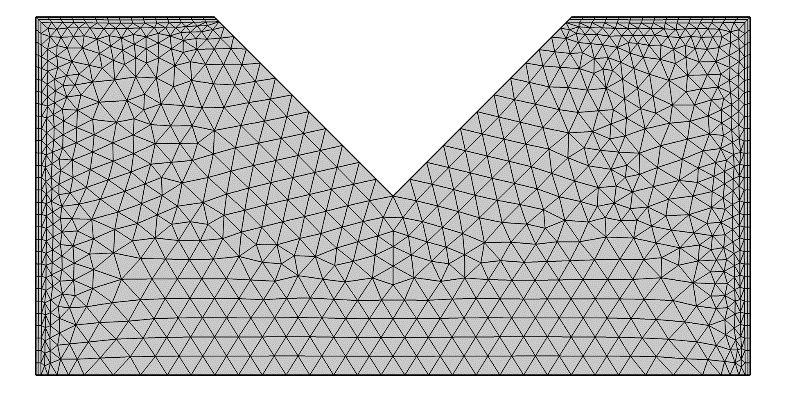


**
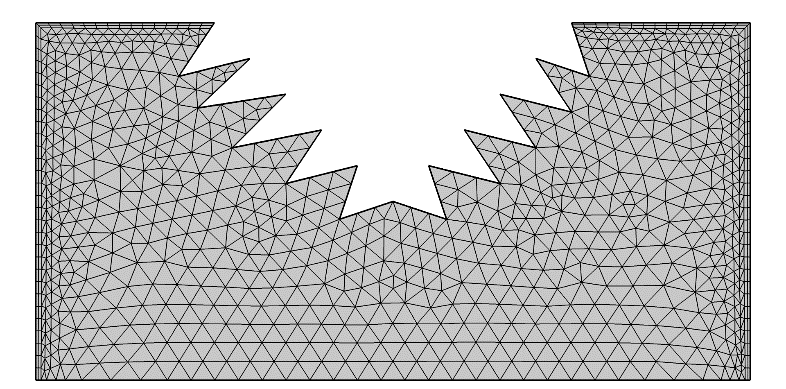
**

The simulation details follow the same approach as described above.

**2.4 The V_oc_ of SPHT**

The Gouy-Chapman model, grounded in the Nernst-Planck equation and Poisson equation (NPP), is employed to explore the hydrovoltaic generation mechanism driven by ion migration in SHIP.^[11]^

The Poisson equation is described as follows:^[11a, 12]^

| $\text{∇}\text{·}\text{(}\text{–}\text{ε}_{\text{0}}\text{ε}_{\text{r}}\text{∇}\text{φ}\text{)}\text{=}\text{F}\sum\text{z}_{\text{i}}\text{C}_{\text{i}}$ | (S13) |
| --- | --- |

where $\text{φ}$, *F*, $\text{ε}$, and *z* were the electric potential, Faraday constant, the dielectric constant valence of ionic species, as well as ion concentration, respectively; $\text{ε}_{\text{0}}$ and $\text{ε}_{\text{r}}$were the permittivity of vacuum and relative permittivity of the medium, respectively; $\text{z}_{\text{i}}$ and $\text{C}_{\text{i}}$were the valency and density of the species, respectively.

Nernst-Planck equation is decribed as follows:^[11a, 12]^

| $\frac{\text{∂}\text{C}_{\text{i}}}{\text{∂t}}\text{=}\text{∇}\text{·}\text{(}\text{D}_{\text{i}}\text{∇}\text{C}_{\text{i}}\text{+}\frac{\text{D}_{\text{i}}\text{C}_{\text{i}}}{\text{K}_{\text{B}}\text{T}}\text{z}_{\text{i}}\text{eφ}\text{)}$ | (S14) |
| --- | --- |
| $j_{i}\text{=}{\text{–}\text{D}}_{\text{i}}\text{(}\text{∇}\text{C}_{\text{i}}\text{+}\frac{\text{z}_{\text{i}}F\text{C}_{\text{i}}}{\text{RT}}\text{∇}\text{φ}\text{)}$ | (S15) |

where *D*, *j*, *R*, and *T* were the ion concentration, diffusion coefficient, ionic flux, ideal gas constant, as well as temperature, respectively; $\text{D}_{\text{i}}$ was the diffusivity of chemical species I; $\text{K}_{\text{B}}$ was Boltzmann’s constant, and *T* was the temperature.

“Electrostatic” and “Transport of Diluted Species” physics within the COMSOL Multiphysics software was used to calculate the electrochemical behavior of all species. The thickness of the Helmholtz layer is taken as the radius of hydrated water and hydronium ions (0.27 nm), and the absolute temperature *T* changes with solar flux. The diffusion coefficients for carriers are assumed to be 1.0 × 10^–15^ m^2^ s^–1^. The effect of solar flux on the potential is converted into reaction boundary conditions.

**3. Supplementary Note 2: Full life cycle analysis**

Life Cycle Assessment (LCA) analysis considers the full process of a product/system from the raw materials extraction stage to product preparation stage, regeneration process during service stage, and end of life, which is a complete “cradle-to-grave” process.^[13]^ As such, the LCA metric was deployed to quantitatively compare the environmental feasibility (*i.e*., the 18 categories of environmental impacts) of our rational-designed SPHT with the benchmark of the traditional industrial sludge drying measure (based on a functional unit of drying 1 t sludge per day) at full life-cycle scale. The calculation process was based on the Simapro^TM^ software and Ecoinvent database, and the analysis followed the International Organization for Standardization (ISO) standard 14040 and 14044 for LCAs and ISO standard 14067 for carbon footprints.

Before the formal LCA analysis, the following assumptions were made:

(1) The default service life of SPHT is 10 years;^[14]^

(2) Each sludge treatment unit can dry 1 t sludge per day (functional unit), so it can dry 3650 t sludge across its whole life cycle;

(3) The sludge drying treatment device works normally during the day, and can be immersed in the regeneration solution at night to achieve regeneration. We suppose that SPHT needs to be regenerated once every 8 days, and it needs to be regenerated 456 times across the sludge drying treatment device’s life cycle. The polypyrrole (PPy) functional layer needs to be reloaded every six months, and it needs to grow 19 times in the whole life cycle;

(4) Waste treatment: the acid generated in the regeneration process during service stage needs to be disposed of as liquid waste. After the sludge drying treatment device is scrapped, the waste is mainly waste wood, which also needs to be scrapped.

| Item | CO_2_ emissions  (t CO_2_-eq/t sludge) | Datebase |
| --- | --- | --- |
| **Stage 1: Raw materials extraction** | | |
| Wood | 5.31E-06 | Sawlog and veneer log, softwood, debarked, measured as solid wood {RoW}\| market for sawlog and veneer log, softwood, debarked, measured as solid wood \| Cut-off, U |
| Deionized water | 2.39E-08 | Water, deionised {RoW}\| market for water, deionised \| Cut-off, U |
| CH_3_COOH | 5.98E-07 | Acetic acid, without water, in 98% solution state {GLO}\| market for acetic acid, without water, in 98% solution state \| Cut-off, U |
| NaClO_2_ | 3.21E-06 | Sodium hypochlorite, without water, in 15% solution state {RoW}\| market for sodium hypochlorite, without water, in 15% solution state \| Cut-off, U |
| Py | 4.27E-08 | Ref.^[6b, 15]^ |
| APS | 1.14E-07 | Ref.^[16]^ |
| **Stage 2: Product preparation** | | |
| Electricity | 1.88E-06 | Electricity, high voltage {GLO}\| market group for electricity, high voltage \| Cut-off, U |
| **Stage 3: Regeneration process during service period** | | |
| Deionized water | 1.09E-05 | Hydrochloric acid, without water, in 30% solution state {RoW}\| market for hydrochloric acid, without water, in 30% solution state \| Cut-off, U |
| HCl | 1.89E-04 | Water, deionised {RoW}\| market for water, deionised \| Cut-off, U |
| Py | 8.11E-07 | Ref.^[6b, 15]^ |
| Electricity | 1.85E-07 | Electricity, high voltage {GLO}\| market group for electricity, high voltage \|  Cut-off, U |
| Acid waste liquid | 6.49E-06 | Wastewater, average {RoW}\| market for wastewater, average \| Cut-off, U |
| **Stage 4: End of Life** | | |
| Waste wood | 4.40E-06 | Waste wood, untreated {RoW}\| market for waste wood, untreated \| Cut-off, U |
| Total | 2.23E-04 |  |

**Note:** (1) Pyrrole (Py) is not available in Ecoinvent database and is therefore referenced the relevant literatures.^[6b, 15]^ (2) Sodium chlorite (NaClO_2_) is not available in Ecoinvent database, and is therefore replaced by a similar salt. According to the correlative reference,^[17]^ the alternative to NaClO_2_ is sodium hypochlorite (NaClO) which is available in the Ecoinvent database. (3) Ammonium persulfate (APS) is also not available in the Ecoinvent database. More specific CO_2_ emission calculation processes about the APS can be found in https://jz.docin.com/p-1450185247.html.

**4. Supplementary Figures and Table**


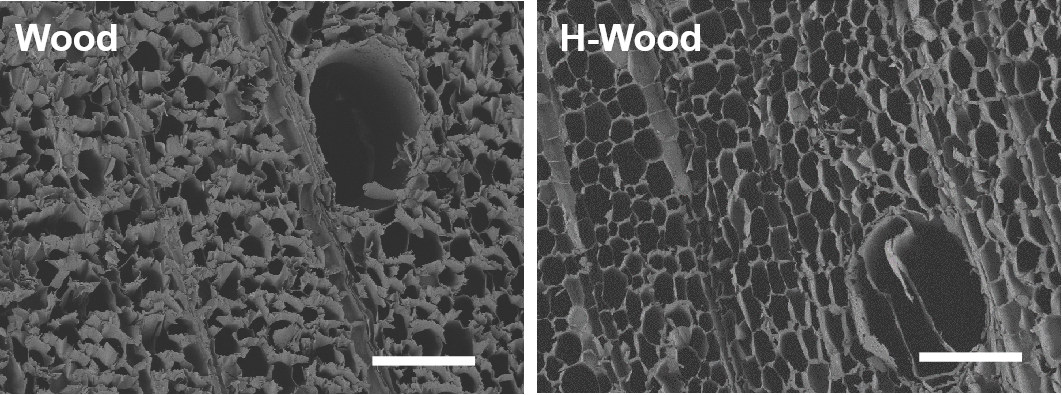


**Figure S1** SEM images of the pristine wood (Wood) and H-Wood. The scale bar is 250 μm.


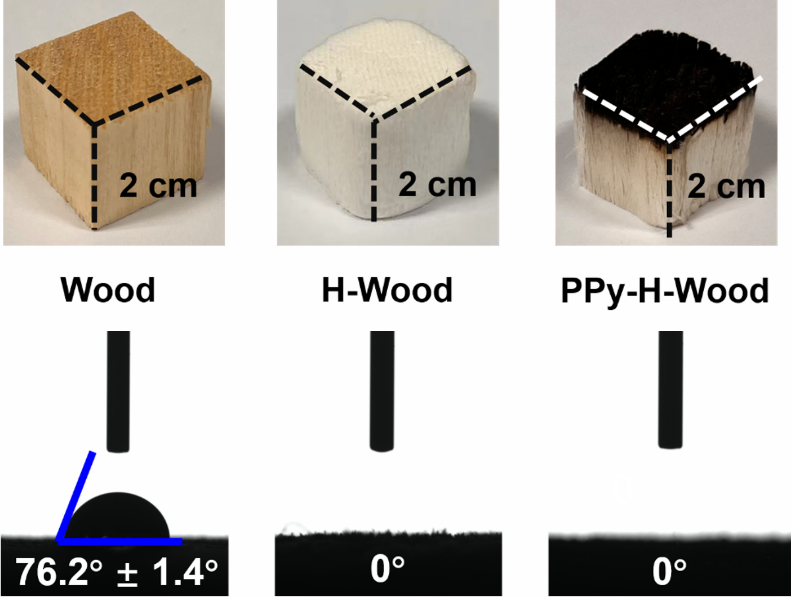


**Figure S2** The digital photos and water contact angles of the three different samples.


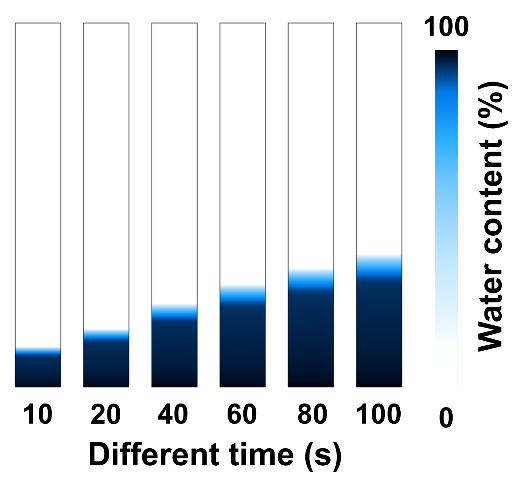


**Figure S3** The water rose in the pristine wood simulated by the COMSOL software.


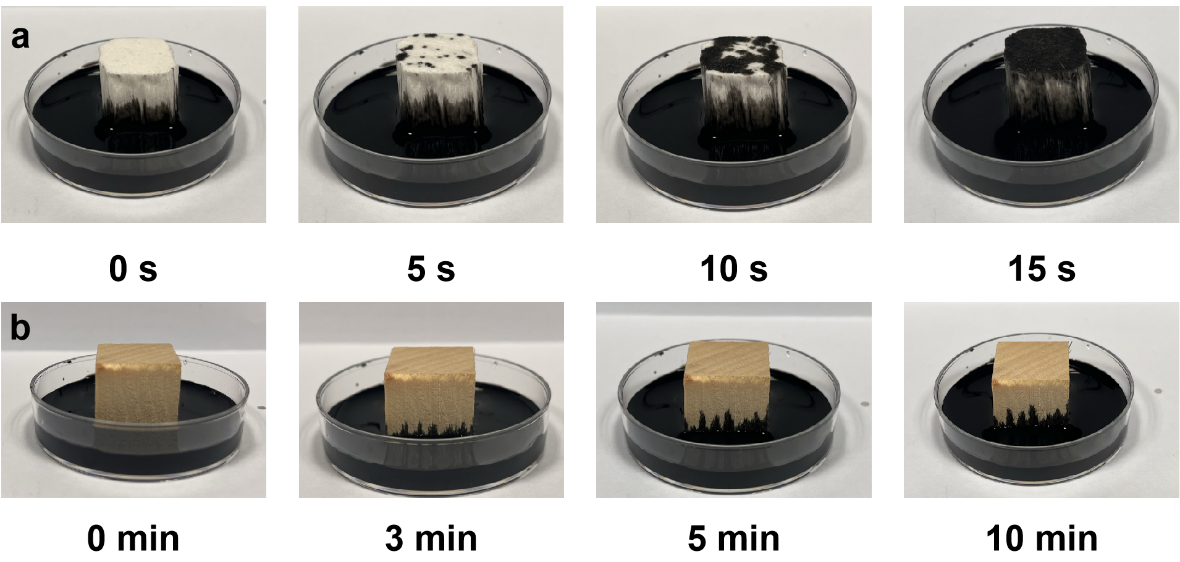


**Figure S4** The digital photos of the ink transport process in (a) H-Wood and (b) pristine wood when the samples were placed in the ink-dyed sludge.


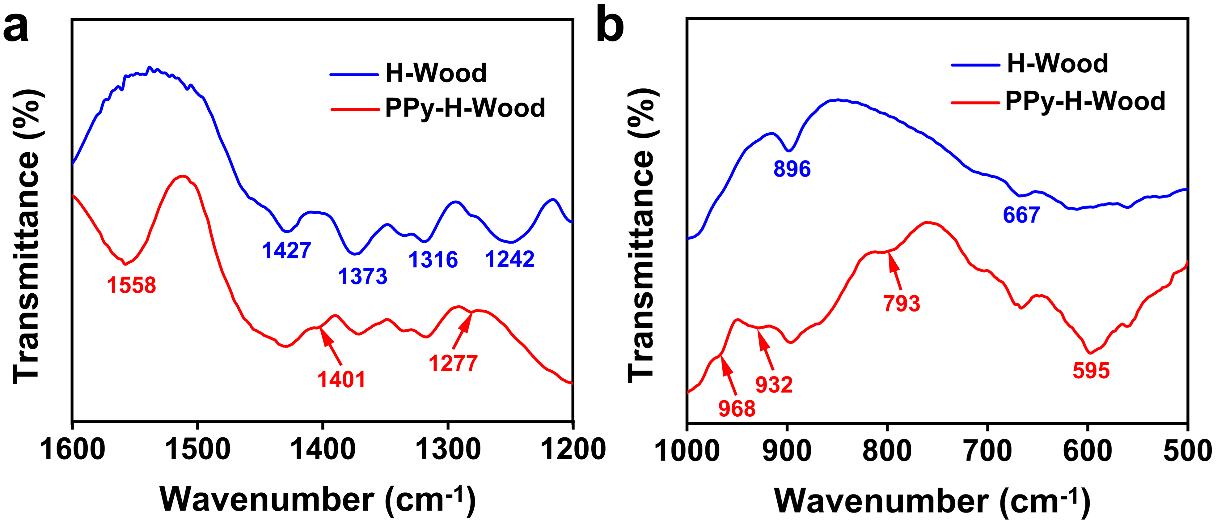


**Figure S5** FTIR spectra of H-Wood and PPy-H-Wood: (a) from 1600 to 1200 cm^−1^; and (b) from 1000 to 500 cm^−1^. After PPy coating, the pristine peaks of H-Wood are weakened or disappear, with newly-appeared peaks. Specifically, the peaks at 1558 and 595 cm^−1^ correspond to the vibrations of the PPy ring.^[18]^ The peaks at 1401 and 1277 cm^−1^ are attributed to the C−N stretching vibration.^[19]^ The peak at 968 cm^−1^ is attributed to the presence of PPy with NH^+^ and N^+^.^[20]^ The peak at 932 cm^−1^ corresponds to the C=C stretching vibration, and the peak at 793 cm^−1^ is related to the C−H vibration.^[21]^


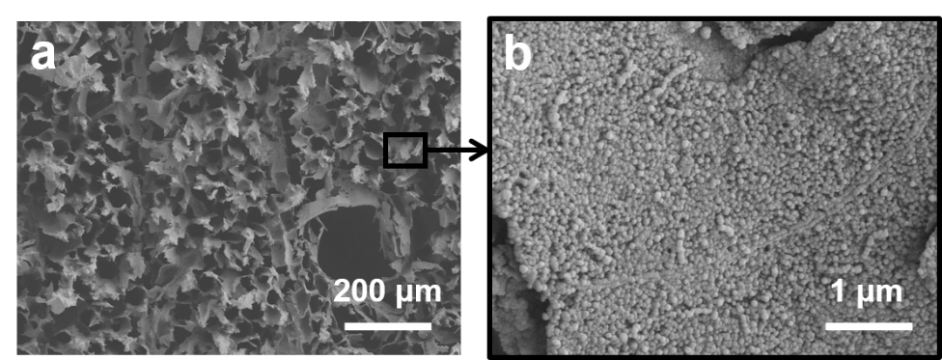


**Figure S6** SEM images of (a) PPy-H-Wood and (b) the fibers in PPy-H-Wood.


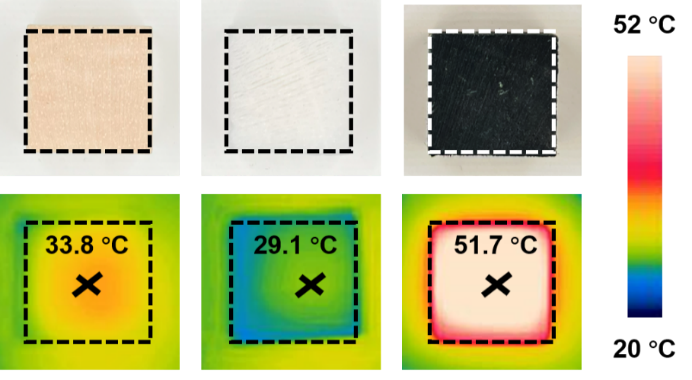


**Figure S7** The digital and IR photos of pristine wood, H-Wood, and PPy-H-Wood under one sun.


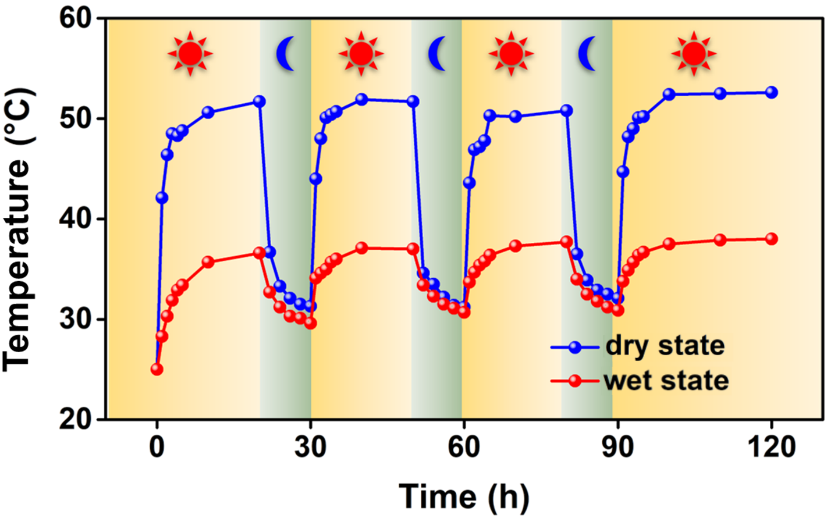


**Figure S8** The surface temperature curves of wet PPy-H-Wood and dry PPy-H-Wood under one sun and in dark conditions.


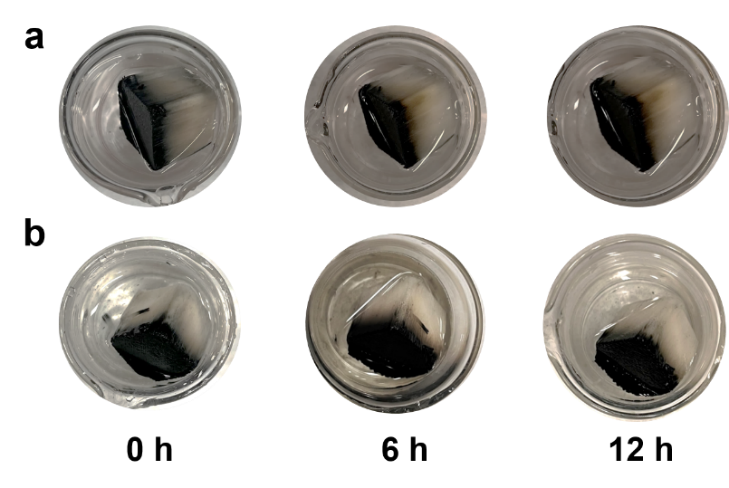


**Figure S9** The digital photos of PPy-H-Wood after ultrasonic treatment in the (a) 0.1 M HCl solution and (b) 0.1 M NaOH solution for 12 h.


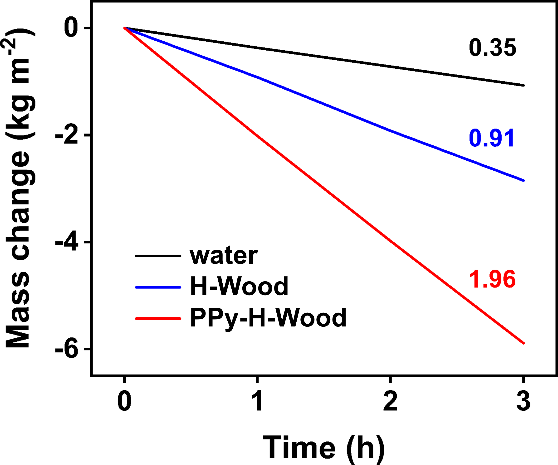


**Figure S10** The mass changes of deionized water, SPHT based on H-Wood, and SPHT based on PPy-H-Wood under one sun. The slope of the curves is the evaporation rate.


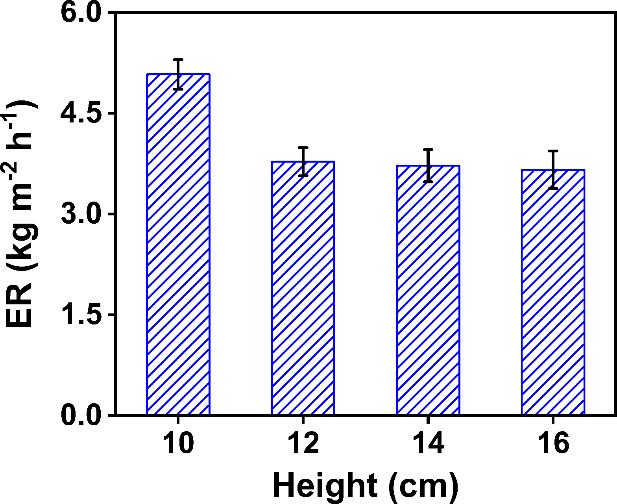


**Figure S11** The evaporation rate of the SPHT based on PPy-H-Wood with different heights under one sun.





**Figure S12** SPHT’s evaporation rate towards treating different water content sludge under one sun.


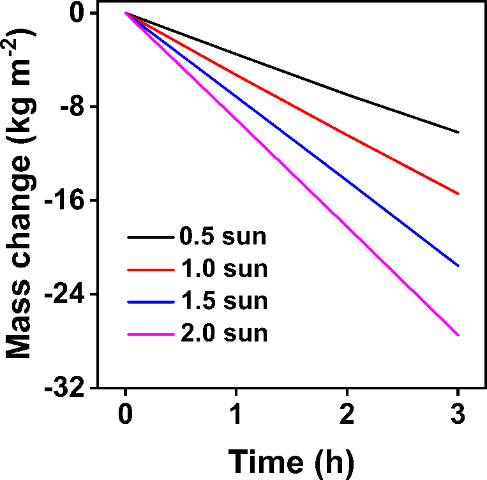


**Figure S13** Mass changes of sludge induced by SPHT under various solar fluxes.


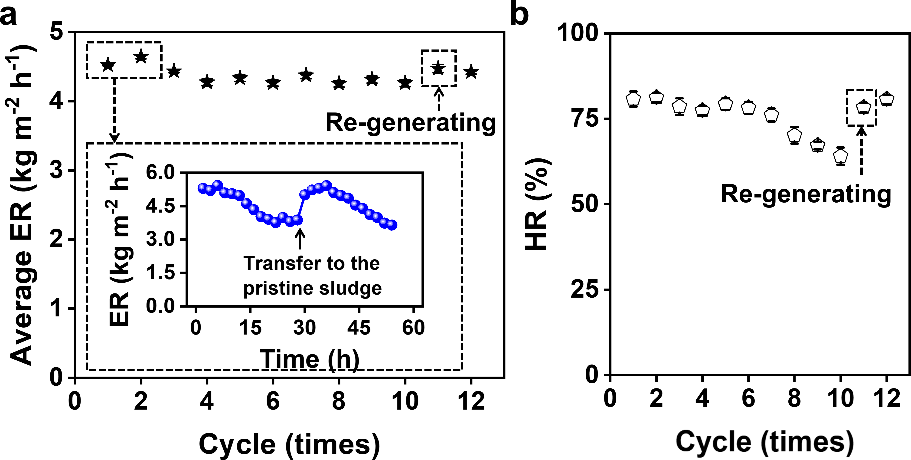


**Figure S14** (a) The average evaporation rate of SPHT in the cycle experiments. Inset: The real-time evaporation rate of SPHT in the first cycle and second cycle; (b) Heavy metal removal ratio (HR) of SPHT during the cycle experiments.


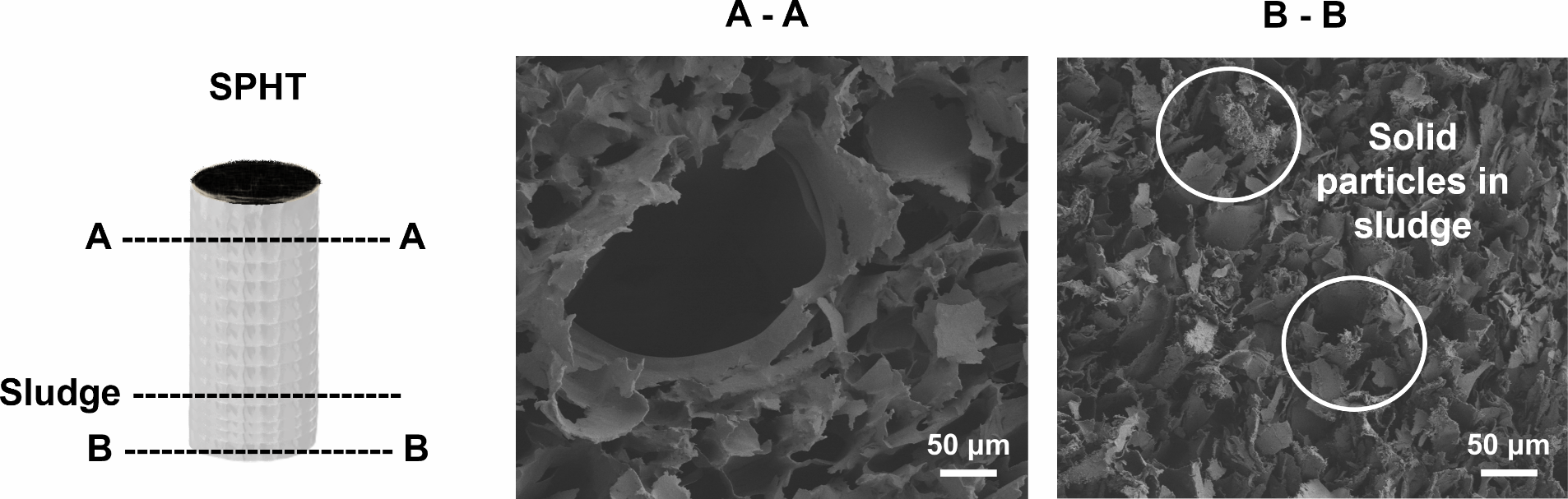


**Figure S15** The SEM images of the SPHT at different locations.


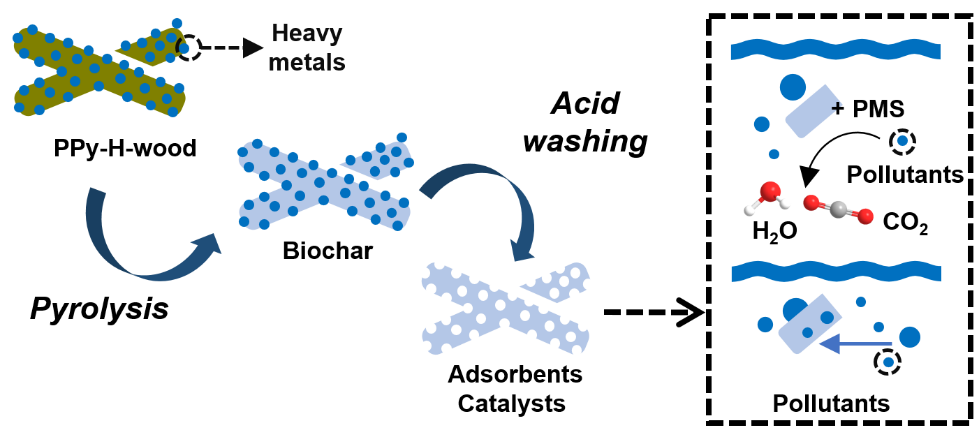


**Figure S16** The potential resource utilization route of the used SPHT after sludge treatment.


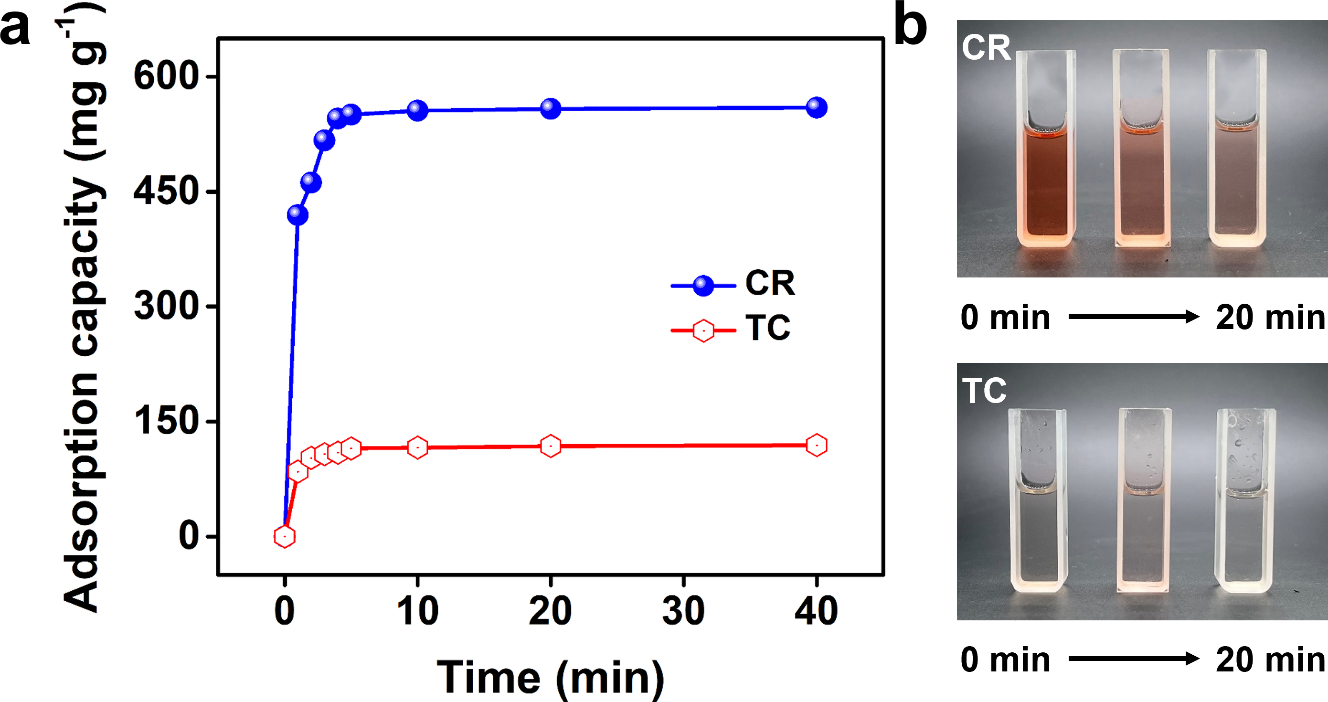


**Figure S17** (a) Adsorption curves of CR and TC by the adsorbents prepared based on the used SPHT. Experimental conditions: [CR]_0_ = 10 mg L^−1^, [TC]_0_ = 0.8 mM, initial *pH* = 6.3. (b) The digital photos of CR and TC during the adsorption process.





**Figure S18** TC removal curves of PMS and PMS together with the catalysts. Experimental conditions: initial *pH* = 6.3, [PMS]_0_ = 0.8 mM, [TC]_-30_ = 10 mg L^−1^.


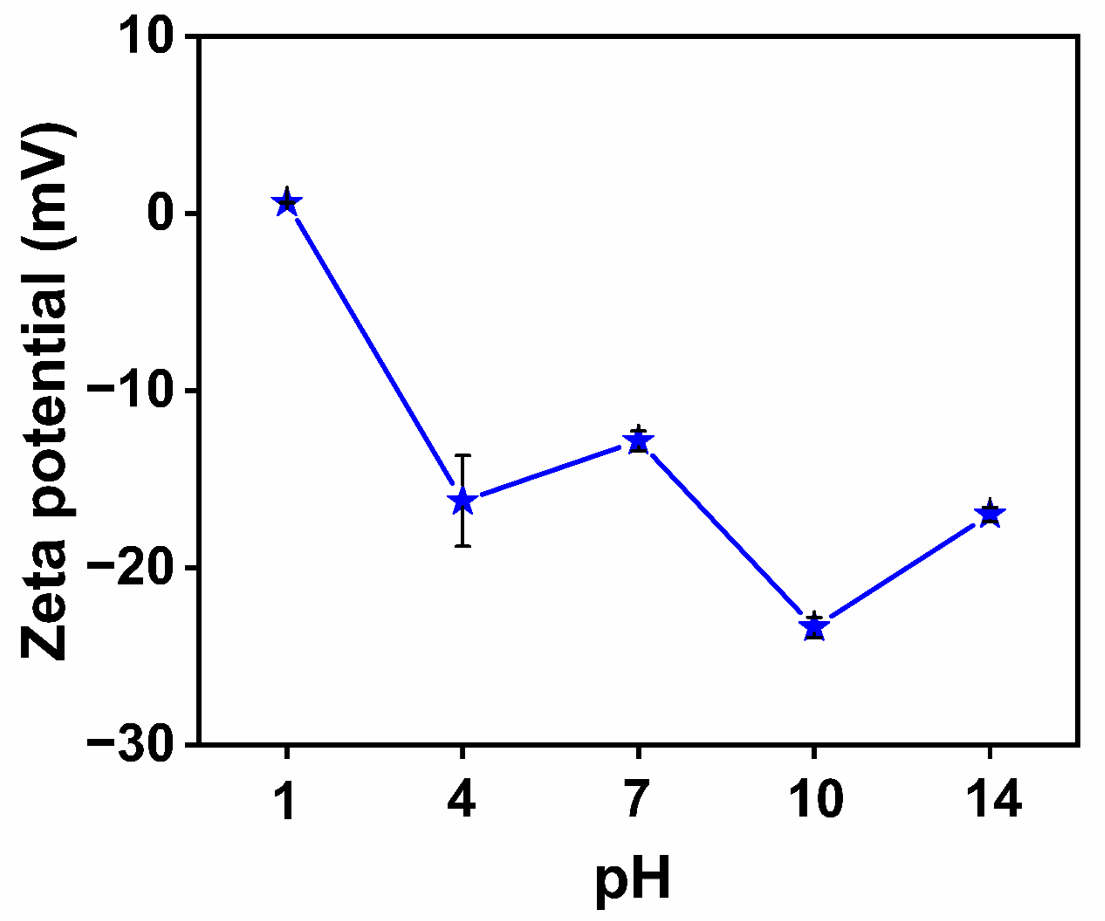


**Figure S19** Zeta potential of the PPy-H-Wood in different pH conditions.


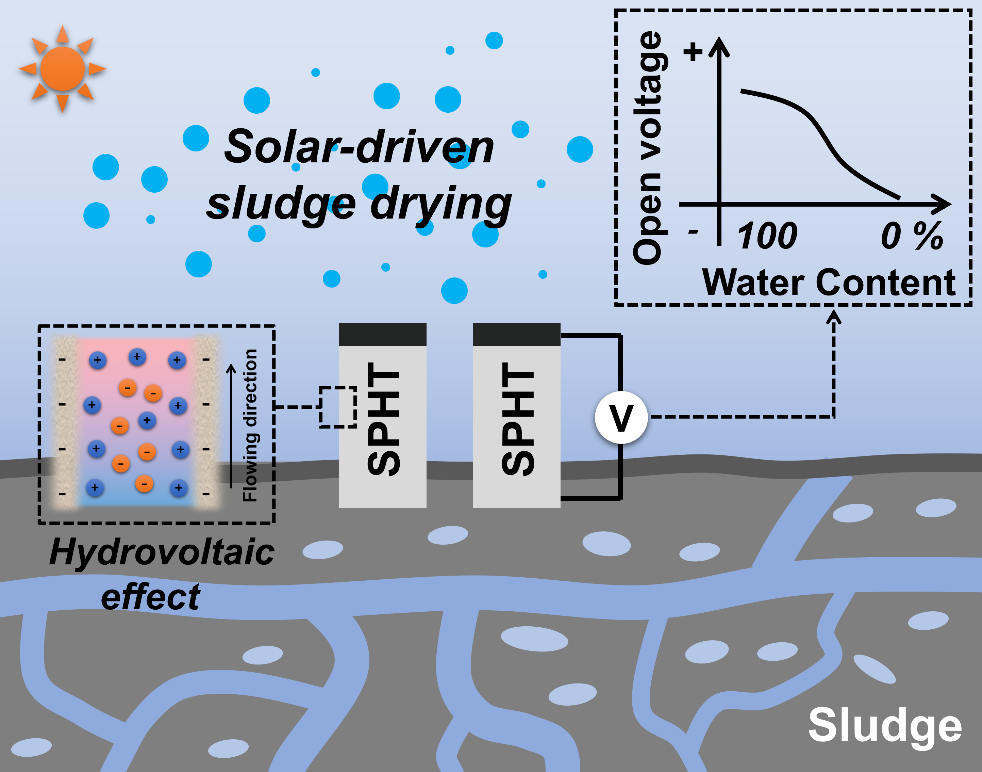


**Figure S20** Schematic illustration for real-time detection of sludge water content during the drying process based on the hydrovoltaic effect.


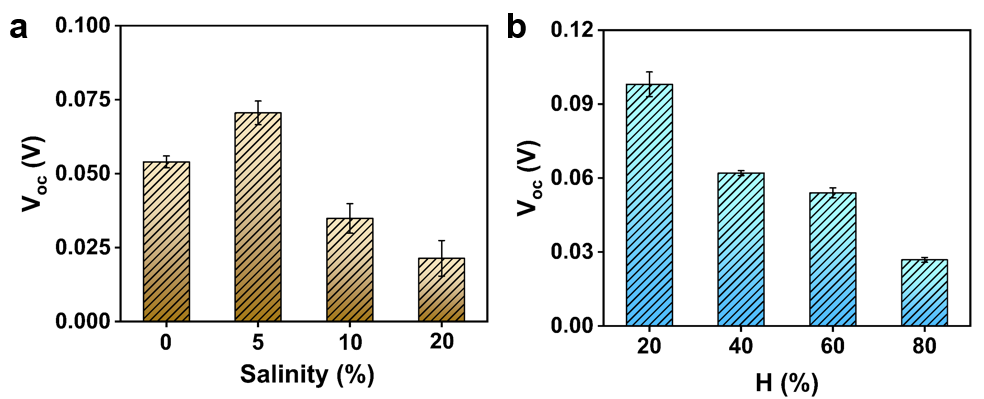


**Figure S21** Open-circuit potential of the SPHT in (a) the different-salinity heavy metal solution (the initial humidity is 33%), and (b) with the different humidity (the initial salinity is 0.1 wt%).


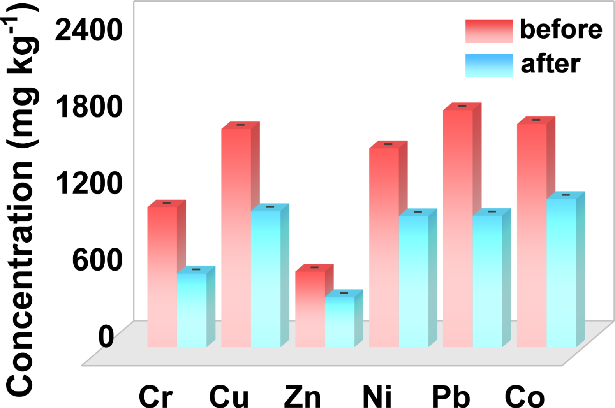


**Figure S22** The heavy metal concentrations of the sludge before and after treatment with SPHT in the dark.


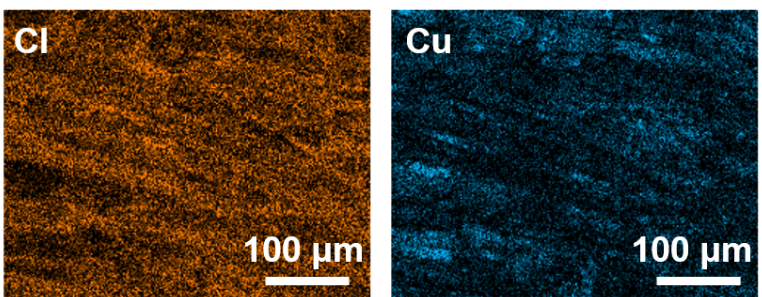


**Figure S23** Elemental mapping images of the SPHT after treating sludge.


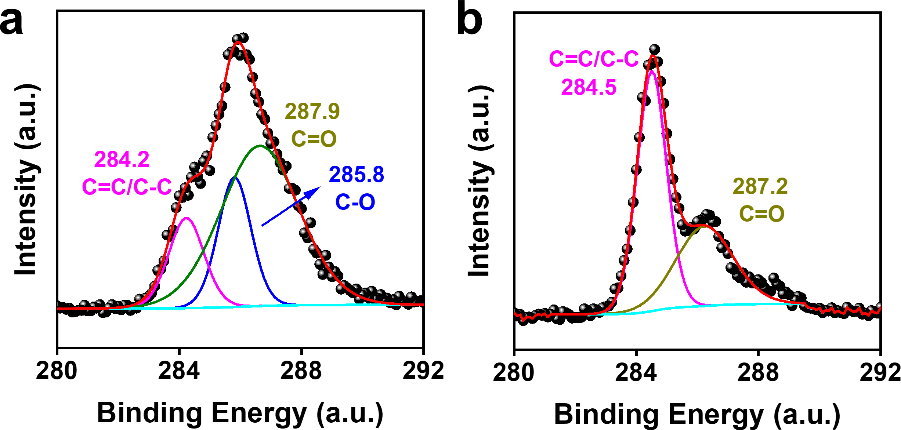


**Figure S24** C1*s* spectra of SPHT (a) before and (b) after Cu^2+^ adsorption.


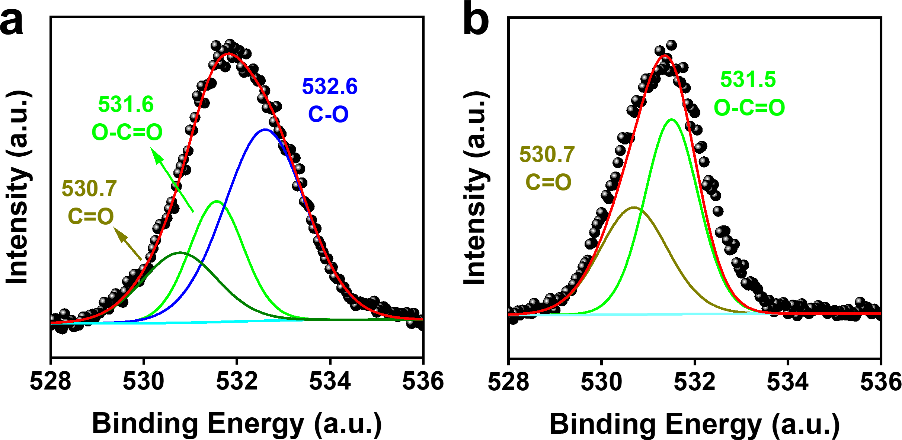


**Figure S25** O1*s* spectra of SPHT (a) before and (b) after Cu^2+^ adsorption.


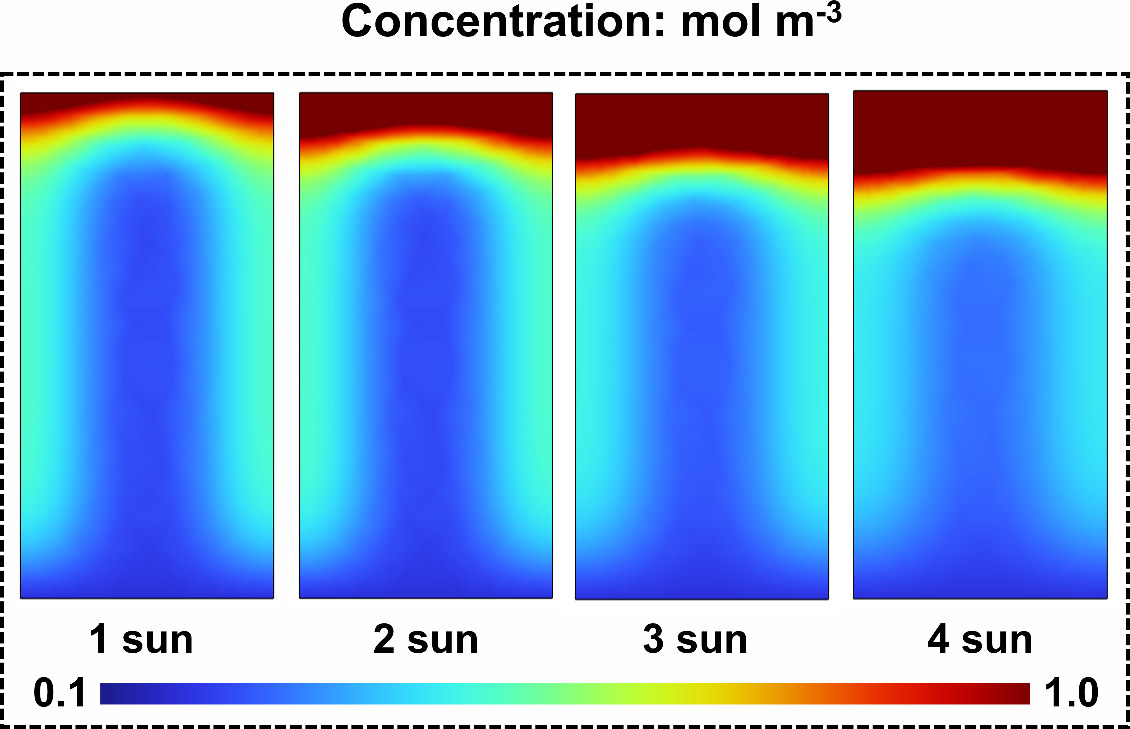


**Figure S26** Concentrations distribution of Cu^2+^ on the channels of SPHT simulated by the COMSOL software under different solar fluxes.


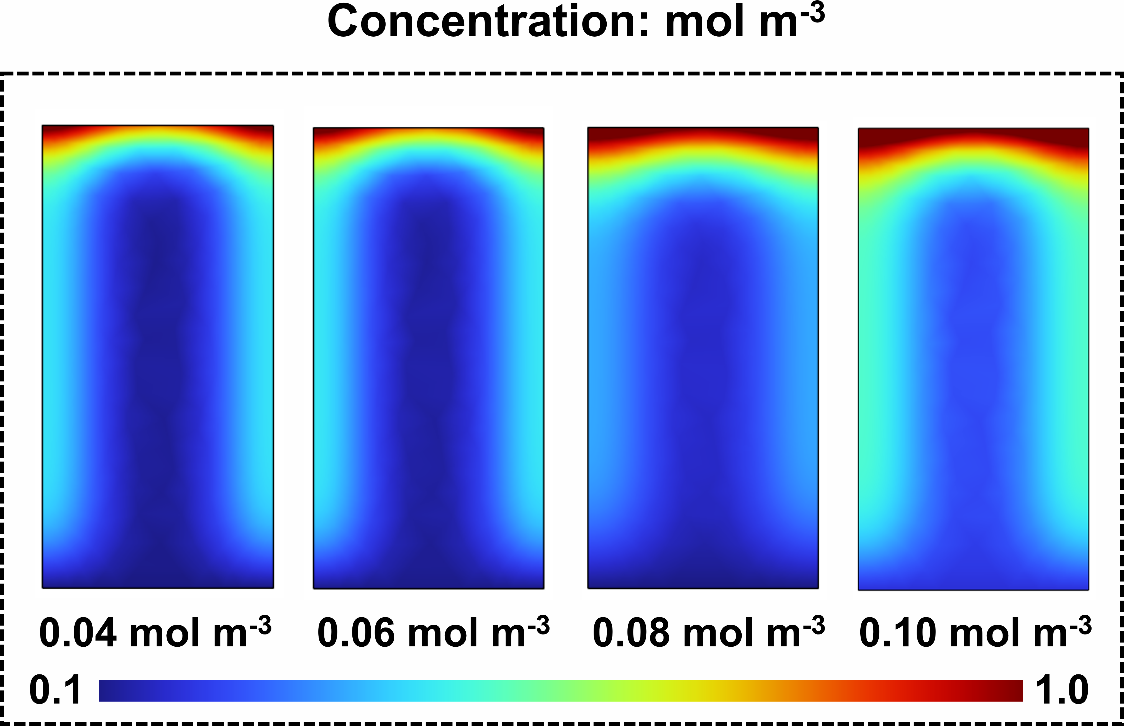


**Figure S27** Concentrations distribution of Cu^2+^ on the channels of SPHT simulated by COMSOL software in the sludge containing different concentrations of Cu^2+^.


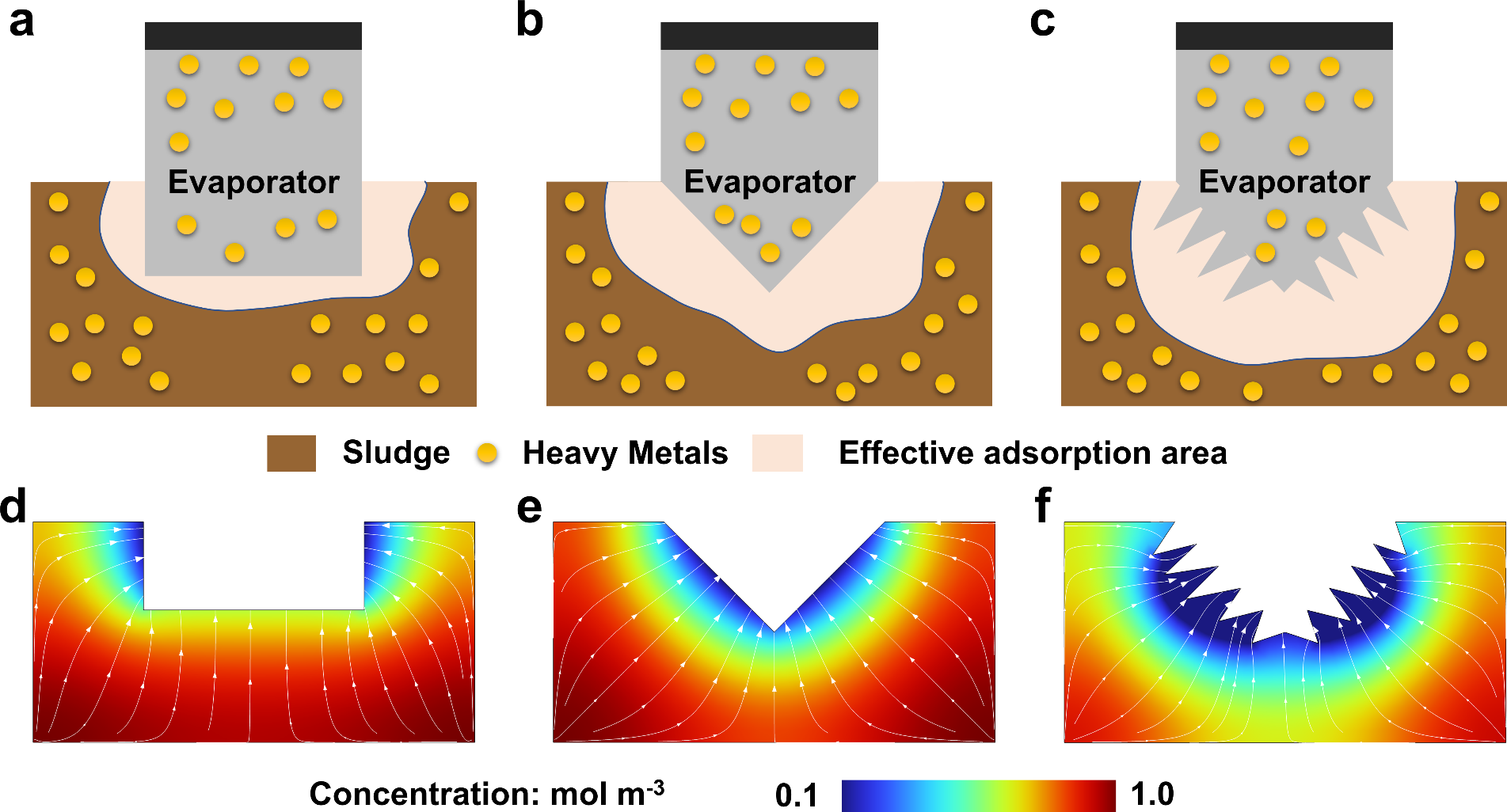


**Figure S28** (a−c) Schematic illustration of the SPHT with different bottom structures. (d−e) Cu^2+^ concentration distribution in the sludge in the different cases of the SPHT featuring different bottom structures, simulated by COMSOL. The effective working fields of a SPHT (a) with a size of 2 cm × 2 cm × 2 cm are about 3.9 cm in diameter and 2.7 cm in depth.


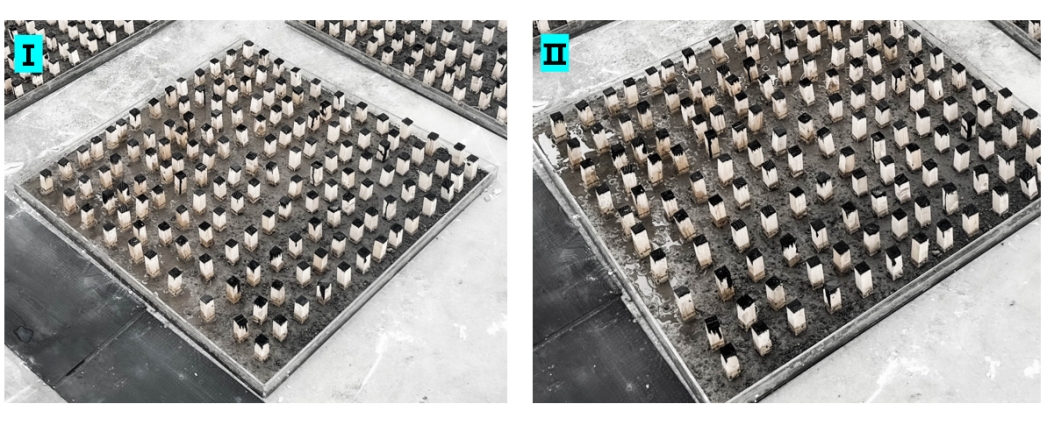


**Figure S29** The digital photos for locations (I) and (II) in the pilot-scale sludge drying device based on SPHT.


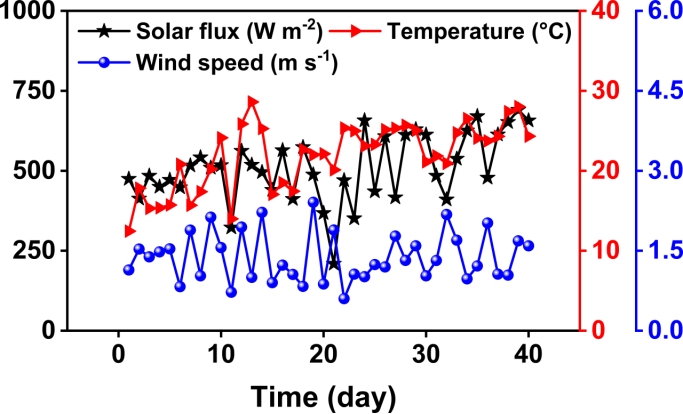


**Figure S30** The weather conditions during the 40-day outdoor experiments.





**Figure S31** Drying ratio and heavy metals removal ratio (HR) of SPHT during the 40-day outdoor experiments.

**Table S1** The evaporation rate of SPHT based on PPy-H-Wood with different immersion depths under one sun. Note that during the above experiment, the evaporation height of the evaporator was always controlled at 8 cm.

| Immersion depth (cm) | Evaporation rate (kg m^–2^ h^–1^) |
| --- | --- |
| 0.5 | 4.37 ± 0.01 |
| 1.0 | 4.85 ± 0.12 |
| 2.0 | 5.13 ± 0.05 |
| 3.0 | 5.04 ± 0.07 |
| 4.0 | 4.92 ± 0.05 |

**Supplementary References**

[1] M. B. Wu, S. Huang, C. Liu, J. Wu, S. Agarwal, A. Greiner, Z. K. Xu, *Journal of Materials Chemistry A* **2020**, 8, 11354.

[2] S. Cheng, Z. Yu, Z. Lin, L. Li, Y. Li, Z. Mao, *Chemical Engineering Journal* **2020**, 401, 126108.

[3] G. Kresse, J. Furthmüller, *Computational Materials Science* **1996**, 6, 15.

[4] J. P. Perdew, K. Burke, M. Ernzerhof, *Physical Review Letters* **1996**, 77, 3865.

[5] K. Zhao, L. X. Zhang, H. Xu, Y. F. Liu, B. Tang, L. J. Bie, *Nanoscale* **2022**, 14, 10980.

[6] a)X. Li, G. Ni, T. Cooper, N. Xu, J. Li, L. Zhou, X. Hu, B. Zhu, P. Yao, J. Zhu, *Joule* **2019**, 3, 1798; b)Z. Yu, R. Gu, Y. Zhang, S. Guo, S. Cheng, S. C. Tan, *Nano Energy* **2022**, 98, 107287; c)Z. Yu, S. Cheng, C. Li, L. Li, J. Yang, *ACS Applied Materials & Interfaces* **2019**, 11, 32038.

[7] Z. Yu, Y. Li, Y. Zhang, P. Xu, C. Lv, W. Li, B. Maryam, X. Liu, S. C. Tan, *Nature Communications* **2024**, 15, 6081.

[8] B. Li, F. Zhou, K. Huang, Y. Wang, S. Mei, Y. Zhou, T. Jing, *Scientific Reports* **2017**, 7, 43082.

[9] a)Z. Yu, S. Cheng, C. Li, Y. Sun, B. Li, *Solar Energy* **2019**, 193, 434; b)Z. Yu, R. Gu, Y. Tian, P. Xie, B. Jin, S. Cheng, *Advanced Functional Materials* **2022**, 32, 2108586; c)Z. Yu, Y. Su, R. Gu, W. Wu, Y. Li, S. Cheng, *Nano-Micro Letters* **2023**, 15, 214; d)L. Li, Y. Su, B. Jin, Z. Yu, S. Cheng, *Separation and Purification Technology* **2024**, 351, 128019; e)S. Xu, K. Zhao, Y. Zhou, K. Zheng, Z. Wang, Z. Yu, N. Cao, X. Liu, *Nano Energy* **2024**, 131, 110232.

[10] J. Xu, Z. Wang, C. Chang, B. Fu, P. Tao, C. Song, W. Shang, T. Deng, *Desalination* **2020**, 484, 114423.

[11] a)D. Lv, S. Zheng, C. Cao, K. Li, L. Ai, X. Li, Z. Yang, Z. Xu, X. Yao, *Energy & Environmental Science* **2022**, 15, 2601; b)T. Xu, X. Ding, C. Shao, L. Song, T. Lin, X. Gao, J. Xue, Z. Zhang, L. Qu, *Small* **2018**, 14, 1704473.

[12] X. Xin, Y. Zhang, R. Wang, Y. Wang, P. Guo, X. Li, *Nature Communications* **2023**, 14, 1759.

[13] a)C. Fu, Z. Wang, Y. Gao, J. Zhao, Y. Liu, X. Zhou, R. Qin, Y. Pang, B. Hu, Y. Zhang, *Nature Sustainability* **2023**, 6, 984; b)R. E. Ciez, J. Whitacre, *Nature Sustainability* **2019**, 2, 148; c)Z. Li, C. Chen, H. Xie, Y. Yao, X. Zhang, A. Brozena, J. Li, Y. Ding, X. Zhao, M. Hong, *Nature Sustainability* **2022**, 5, 235.

[14] C. Dang, H. Wang, Y. Cao, J. Shen, J. Zhang, L. Lv, G. Xu, M. Zhu, *Energy & Environmental Science* **2022**, 15, 5405.

[15] S. Xu, Y. Zhao, S. Jiao, Z. Wang, Z. Yu, C. Sun, X. Liu, *Advanced Science* **2024**, 2400856.

[16] https://jz.docin.com/p-1450185247.html/

[17] R. Rai, R. Ranjan, P. Dhar, *Science of The Total Environment* **2022**, 846, 157301.

[18] a)A. Thakur, B. Lokhande, *Journal of Materials Science: Materials in Electronics* **2018**, 29, 1630; b)R. Kandulna, R. Choudhary, R. Singh, *Journal of Inorganic and Organometallic Polymers and Materials* **2019**, 29, 730.

[19] F. Memioğlu, A. Bayrakçeken, T. Öznülüer, M. Ak, *International Journal of Hydrogen Energy* **2012**, 37, 16673.

[20] Y. Wang, J. Yang, L. Wang, K. Du, Q. Yin, Q. Yin, *ACS Applied Materials & Interfaces* **2017**, 9, 20124.

[21] Q. Wang, Y. Deng, J. Chen, L. Lu, Y. Ma, L. Zang, *Journal of Alloys and Compounds* **2022**, 927, 167117.
